# O-GlcNAcylation licenses RNF166 to degrade the M protein of porcine coronaviruses

**DOI:** 10.1371/journal.ppat.1014301

**Published:** 2026-06-25

**Authors:** Runhui Qiu, Yucheng Zhang, Junwei Zhou, Jianan Wang, Wenbing Tang, Xinliang Xie, Yan Cheng, Tong Ding, Peng Sun, Yuting Shi, Caili Xi, Yanrong Zhou, Liurong Fang, Shaobo Xiao

**Affiliations:** 1 National Key Laboratory of Agricultural Microbiology, College of Veterinary Medicine, Huazhong Agricultural University, Wuhan, China; 2 Key Laboratory of Preventive Veterinary Medicine in Hubei Province, Cooperative Innovation Center for Sustainable Pig Production, Wuhan, China; Guangzhou National Laboratory, CHINA

## Abstract

Uridine diphosphate N‑acetylglucosamine (UDP‑GlcNAc) has often been overlooked because its source pathway contributes little to glucose flux. However, through O‑GlcNAcylation, even small fluctuations in UDP‑GlcNAc levels can be amplified to shape immune responses. In this study, we utilized porcine deltacoronavirus (PDCoV), an emerging enteropathogenic coronavirus with zoonotic potential, as a model to investigate the role of UDP-GlcNAc in viral infection. Our findings demonstrate that upon PDCoV infection, host cells increase the synthesis of UDP-GlcNAc, which inhibits viral replication by remodeling metabolic pathways. Mechanistically, O-linked N-acetylglucosamine transferase (OGT) transfers an O-GlcNAc moiety from UDP-GlcNAc to RNF166 at T157, resulting in O-GlcNAcylation. This modification enables RNF166 to ubiquitinate the PDCoV membrane (M) protein at K207, thereby promoting its degradation via the ubiquitin-proteasome pathway. Notably, these effects are common in the host response to porcine coronavirus infections, highlighting the intricate interplay among metabolism, glycosylation, and ubiquitination in immune responses.

## Author summary

The interplay between cellular metabolism and antiviral defense is a rapidly evolving field of great significance. Using the emerging zoonotic coronavirus PDCoV as a model, we show that virus infection elevates cellular levels of UDP-GlcNAc. An O-GlcNAc moiety derived from this metabolite is transferred by OGT to the E3 ubiquitin ligase RNF166 at threonine 157, inducing its O-GlcNAcylation and enabling RNF166 to target a conserved lysine residue in the M protein of porcine coronaviruses for proteasomal degradation, thereby limiting viral replication. These findings identify a metabolic O-GlcNAcylation axis that strengthens host defense against porcine coronaviruses.

## Introduction

Metabolic intervention serves as a defense strategy for immunity by restricting viral resources and replication through modulation of the host’s metabolism [1,2]. The hexosamine biosynthetic pathway (HBP), a minor pathway in glucose metabolism, converts glucose into uridine diphosphate N-acetylglucosamine (UDP-GlcNAc) at a rate of only 3% to 5% [3,4]. Due to these low conversion rates, the HBP has received less attention in metabolic intervention studies, where glycolysis is typically prioritized [5–7]. However, UDP-GlcNAc, the end product of the HBP, functions as a substrate for protein O-GlcNAcylation. O-GlcNAc transferase (OGT) catalyzes the transfer of the O-GlcNAc moiety from UDP-GlcNAc to serine (S) or threonine (T) residues on substrate proteins [8]. This mechanism enables even a minimal quantity of UDP-GlcNAc to significantly amplify biological functions through O-GlcNAcylation, including the regulation of protein localization, activity, and stability, as well as the integration of metabolic networks involving amino acids, fatty acids, and nucleotides [3,8,9]. Therefore, the significance of UDP-GlcNAc in host metabolic interventions may have been severely underestimated.

Currently, the role of O-GlcNAcylation during viral infections remains a topic of considerable debate [10,11]. On one hand, some studies propose that O-GlcNAcylation acts as a powerful host weapon, inhibiting viral gene synthesis through effector molecules such as mitochondrial antiviral signaling protein (MAVS), interferon regulatory factor 5 (IRF5), and sterile alpha motif and HD domain-containing protein 1 (SAMHD1) [12–15]. On the other hand, this modification is also perceived as a ‘booster’ for viruses, as it facilitates viral replication and survival by stabilizing viral genome structures and disrupting TNF Receptor-Associated Factor 3 (TRAF3)-mediated activation of the IRF3 signaling pathway [16–18]. Moreover, O-GlcNAcylation has been demonstrated to enhance viral fitness by ensuring the stability of the SARS-CoV-2 spike (S) protein, which is crucial for the virus’s entry into host cells [19–21]. The dual roles of O-GlcNAcylation complicate our understanding of its function during viral infections, making it challenging to define its therapeutic potential [11]. Consequently, this ambiguity presents significant challenges for research on the immunological roles of HBP and the development of targeted therapies for emerging pathogens.

Coronaviruses (CoVs), the members of the subfamily *Orthocoronavirinae* within the family *Coronaviridae*, are widely distributed among humans, other mammals, and birds, causing a variety of diseases, including respiratory, intestinal, hepatic, and neurological disorders [22]. Based on genomic sequences, the subfamily *Orthocoronavirinae* can be classified into four genera: *Alphacoronavirus* (α‐CoV), *Betacoronavirus* (β‐CoV), *Gammacoronavirus* (γ-CoV), and *Deltacoronavirus* (δ‐CoV) [22,23]. Generally, α‐CoVs and β‐CoVs primarily infect mammals, γ-CoVs infect birds, while δ‐CoVs can infect both mammals and birds [24,25]. Porcine deltacoronavirus (PDCoV) is an emerging enteropathogenic coronavirus that was first identified in Hong Kong in 2012, with detailed pathogen characterization conducted in the United States in 2014 [26,27]. Clinically, PDCoV infection is associated with frequent diarrhea, vomiting, dehydration, and high mortality rates in piglets [26]. Notably, PDCoV exhibits significant potential for cross-species transmission, with documented infections in various mammals, including calves, poultry, and mice [28,29]. Recently, PDCoV infections have also been detected in plasma samples from three Haitian children with acute febrile illness [30]. As the eighth coronavirus capable of infecting humans, PDCoV represents a shift from an issue primarily confined to agriculture to a profound global public health challenge [31]. Consequently, PDCoV serves as an appropriate model for studying the interplay between coronaviruses and hosts.

In this study, we employed PDCoV as a model to demonstrate that UDP-GlcNAc mediates host antiviral defense via O-GlcNAcylation. During this process, the RING finger protein 166 (RNF166) is endowed with the capacity to ubiquitinate and degrade the viral membrane (M) protein, effectively restricting viral replication. Importantly, this mechanism appears to be conserved among porcine coronaviruses, while not for other coronaviruses, highlighting a host-restricted yet previously unrecognized antiviral strategy.

## Results

### PDCoV infection induces intracellular UDP-GlcNAc accumulation

**Fig 1:**
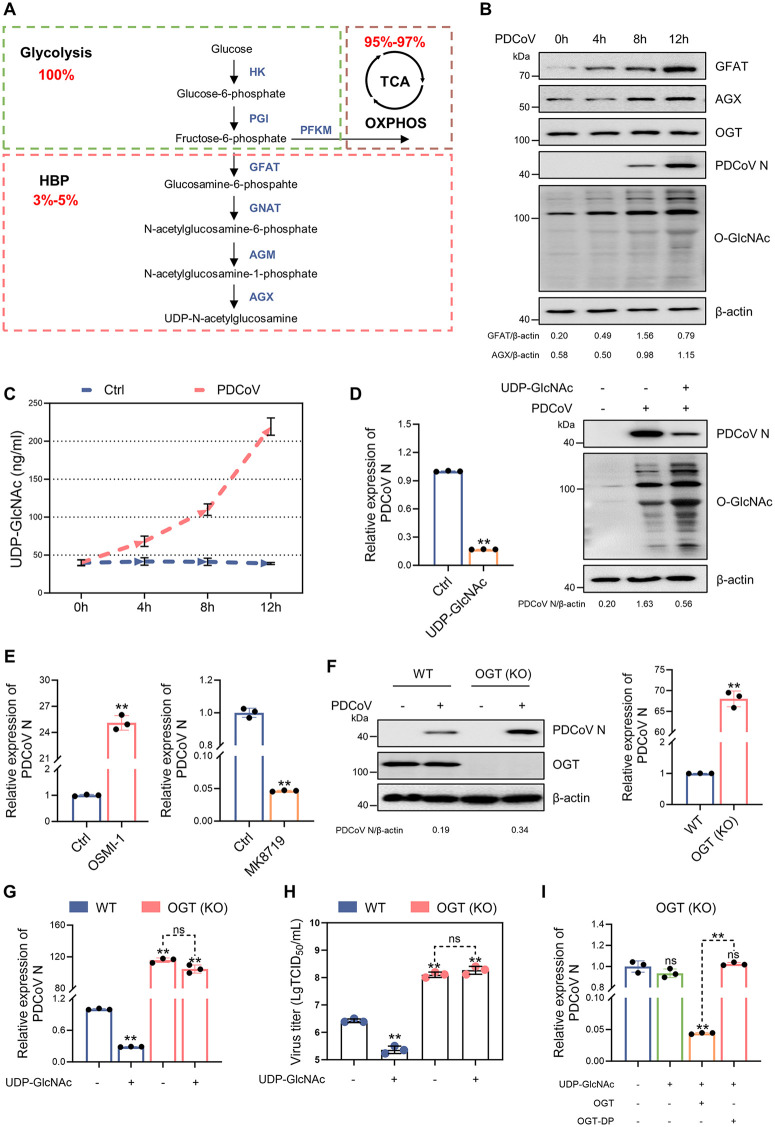
UDP-GlcNAc inhibits PDCoV replication via O-GlcNAcylation. **A,** Schematic diagram of the hexosamine biosynthetic pathway (HBP) from glycolysis to UDP-GlcNAc. **B–C**, IPI-2I cells were infected with PDCoV (MOI = 2) and harvested at the indicated time points for HBP flux analysis. **(B)** Protein levels of GFAT, AGX, OGT, PDCoV N, and cellular O-GlcNAcylation, assessed by western blot. Ratios below the images represent the protein levels of GFAT and AGX relative to β-actin, as quantified by the ImageJ software. **(C)** Intracellular UDP-GlcNAc content, measured by ELISA. **D-I,** IPI‑2I cells were subjected to the indicated treatments, infected with PDCoV (MOI = 2), and harvested at 12 h post-infection (hpi) for viral replication analysis. (D) Cells supplemented with or without UDP‑GlcNAc (32 mM). Ratios below the images represent the protein levels of PDCoV relative to β-actin, as quantified by the ImageJ software. **(E)** Cells treated with the O‑GlcNAcylation agonist MK‑8719 (10 μM) or inhibitor OSMI‑1 (50 μM). **(F)** Wild-type versus OGT knockout (KO) IPI-2I cells. Ratios below the images represent the protein levels of PDCoV relative to β-actin, as quantified by the ImageJ software. **(G–H)** Wild‑type and OGT (KO) IPI-2I cells supplemented with or without UDP‑GlcNAc. **(I)** OGT (KO) IPI-2I cells overexpressing OGT or catalytically inactive OGT‑DP, with UDP‑GlcNAc supplementation. β-actin and GAPDH served as internal controls for western blot and RT-qPCR, respectively. Data in panels C, D, E, F, G, H, and I represent mean ± s.d. (n = 3). Statistical significance was determined by two-tailed Student’s t-test; *P < 0.05; **P < 0.01; ns, not significant.

To investigate whether UDP-GlcNAc contributes to host antiviral defense, IPI-2I cells, an immortalized porcine intestinal epithelial cell line, were infected with PDCoV. Subsequently, the HBP flux, as the primary source of UDP-GlcNAc[3, 5, 32], was examined at various time points post-infection. Specifically, we assessed metabolic activity by quantifying the expression levels of glutamine–fructose-6-phosphate amidotransferase (GFAT), amino sugar-1-phosphate N-acetyltransferase (AGX), and intracellular UDP-GlcNAc, which correspond to pathway initiation, completion, and output, respectively (Fig 1A). Our results revealed that PDCoV infection progressively increased GFAT and AGX expression at both mRNA and protein levels (Figs 1B and S1A). As a result, this upregulation of rate-limiting enzymes, indicative of enhanced HBP flux, directly drove the accumulation of intracellular UDP-GlcNAc (Fig 1C).

### UDP-GlcNAc inhibits PDCoV replication via O-GlcNAcylation

We hypothesized that the increase in UDP-GlcNAc following PDCoV infection reflects a metabolic adaptation of the host immune response aimed at restricting viral replication. To test this hypothesis, non-cytotoxic levels of UDP-GlcNAc were supplemented to IPI-2I cells during infection (S1B Fig). As shown in Figs 1D and S1C, UDP-GlcNAc supplementation effectively inhibited PDCoV replication. Notably, supplementation with UDP-GlcNAc was accompanied by increased global protein O-GlcNAcylation (Fig 1D), suggesting that UDP-GlcNAc may contribute to host antiviral defense through this modification. To this end, IPI-2I cells were treated with OSMI-1 (an O-GlcNAcylation inhibitor) or MK-8719 (an O-GlcNAcylation activator) to modulate cellular O-GlcNAcylation, followed by PDCoV infection. Our results showed that OSMI-1 markedly promoted viral replication, while MK-8719 exhibited the opposite effect (Figs 1E and S1D-S1E). In addition, overexpression of O-GlcNAcase (OGA), which removes O-GlcNAcylation from proteins, also promoted PDCoV replication (S1F Fig). In summary, these findings suggest that UDP-GlcNAc may inhibit PDCoV replication by enhancing protein O-GlcNAcylation.

Given that O-GlcNAcylation is catalyzed exclusively by OGT, we generated OGT-knockout (KO) IPI-2I cells using CRISPR/Cas9-mediated gene editing. As expected, OGT deficiency markedly enhanced PDCoV replication (Figs 1F and S1G). Furthermore, UDP-GlcNAc supplementation also failed to limit viral replication in the absence of OGT (Fig 1G-1H), indicating that OGT is indispensable for the anti-PDCoV effect of UDP-GlcNAc. To further confirm this, we constructed a catalytically inactive OGT double mutant (OGT-DP), which is unable to transfer an O-GlcNAc moiety from UDP-GlcNAc to substrate proteins [32,33]. Compared to wild-type OGT (OGT-WT), OGT-DP exhibited a significantly reduced ability to limit PDCoV replication (S1H-S1I Fig). Subsequently, we detected the replication of PDCoV in OGT (KO) cells supplemented with sufficient UDP-GlcNAc and overexpressing either OGT-WT or OGT-DP. The results showed that only overexpression of OGT-WT effectively suppressed PDCoV replication, while OGT-DP failed to inhibit viral replication (Fig 1I). Collectively, these findings suggest that elevated cellular UDP-GlcNAc during PDCoV infection constitutes a metabolic component of the antiviral response to restrict PDCoV replication, with UDP-GlcNAc mediating anti-PDCoV effects through OGT-catalyzed O-GlcNAcylation of target proteins.

### Identification of host O-GlcNAcylation targets following PDCoV infection

**Fig 2:**
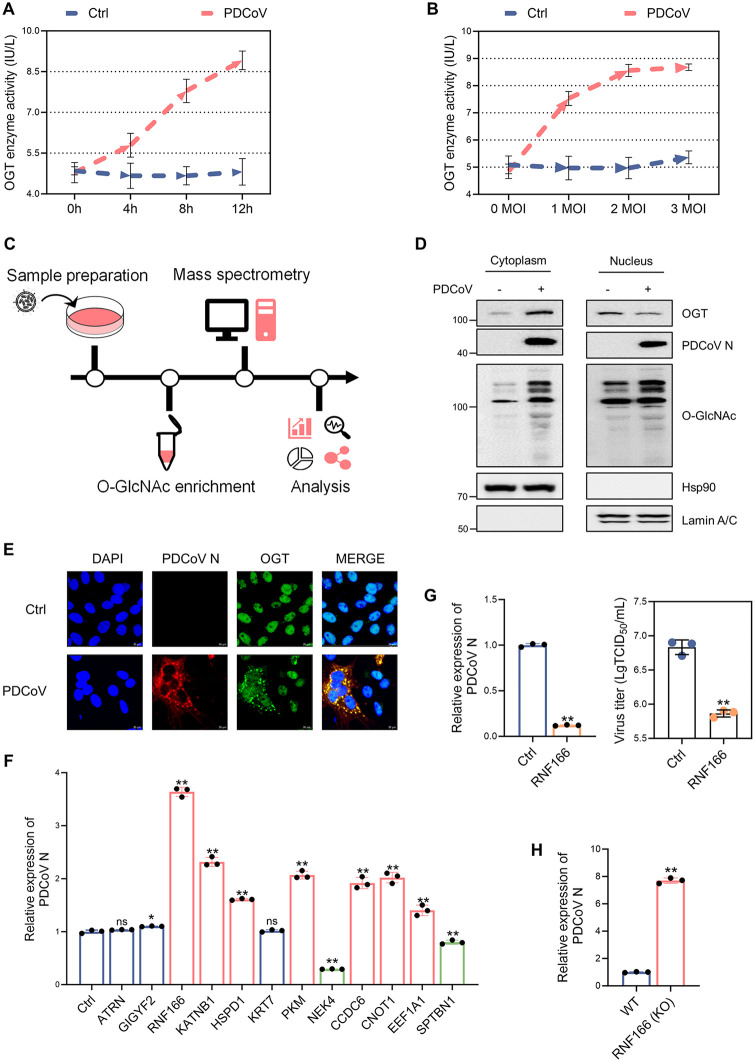
RNF166 inhibits the replication of PDCoV. **A–B**, OGT enzymatic activity in IPI-2I cells after PDCoV infection at the indicated times (MOI = 2) (A) or at 12 hpi with various MOIs **(B)**. **C,** Schematic diagram of O-GlcNAc–focused proteomics under PDCoV infection conditions. **D–E**, OGT localization in PDCoV-infected IPI-2I cells assessed by nuclear and cytoplasmic fractionation assay (D) and indirect immunofluorescence assay **(E)**. **F**, PDCoV replication in IPI-2I cells transfected with specific siRNAs targeting the indicated host proteins, analyzed by RT‑qPCR. **G**, Viral replication in RNF166-overexpressing cells measured by RT-qPCR and TCID₅₀ assay. **H**, PDCoV replication compared between wild-type and RNF166 (KO) IPI-2I cells. Data in panels A, B, F, G and H represent mean ± s.d. (n = 3). Statistical significance was determined by two-tailed Student’s t-test; *P < 0.05; **P < 0.01; ns, not significant.

To identify the protein targets mediated by OGT that underlie the antiviral effects of UDP-GlcNAc, we first aimed to establish infection conditions that would robustly induce O-GlcNAcylation in host cells for subsequent proteomic analyses. The results revealed that host cells exhibited increased O-GlcNAcylation in response to PDCoV infection in a time- and dose-dependent manner (Figs 1B and S2A). Notably, this effect was not attributable to increased expression of OGT, but rather to its enzymatic activity (Fig 2A-2B). Furthermore, a total of 105 proteins and 166 targets of O-GlcNAcylation following PDCoV infection were identified through proteomic profiling (Fig 2C). Enrichment analysis indicated that the targets of O-GlcNAcylation are involved in critical biological processes, including cellular metabolism, signal transduction, and gene regulation (S3A-C Fig). Importantly, we observed that OGT translocated from the nucleus to the cytoplasm following PDCoV infection (Figs 2D-E and S2B), as demonstrated by nuclear–cytoplasmic fractionation and immunofluorescence analyses. Correspondingly, cytoplasmic O-GlcNAcylation rose markedly, while nuclear levels changed little, suggesting that newly O-GlcNAcylated cytoplasmic proteins, rather than nuclear proteins, play a crucial role in restricting viral replication.

### RNF166 inhibits the replication of PDCoV

In light of the aforementioned findings, the 105 O-GlcNAcylated proteins were cross-referenced with the Human Protein Atlas (HPA) database, and 12 candidates exclusively localized to the cytoplasm were selected (S5 Table). Then, a preliminary antiviral screen was conducted by transfecting siRNAs targeting these 12 O-GlcNAcylated cytoplasmic proteins individually. Our results showed that silencing RNF166 had the most pronounced effect on promoting PDCoV replication (Fig 2F). RNF166, an E3 ubiquitin ligase, is known to regulate multiple cellular signaling pathways [34,35]. We further confirmed its critical role in anti-PDCoV activity through the transient transfection of a eukaryotic RNF166 expression construct and the generation of RNF166-knockout IPI-2I cell lines using CRISPR/Cas9-mediated gene editing (Figs 2G-2H and S2C-S2E). Therefore, RNF166 was selected for further investigation in subsequent experiments.

### PDCoV infection increases O-GlcNAcylation of RNF166 at T157

**Fig 3:**
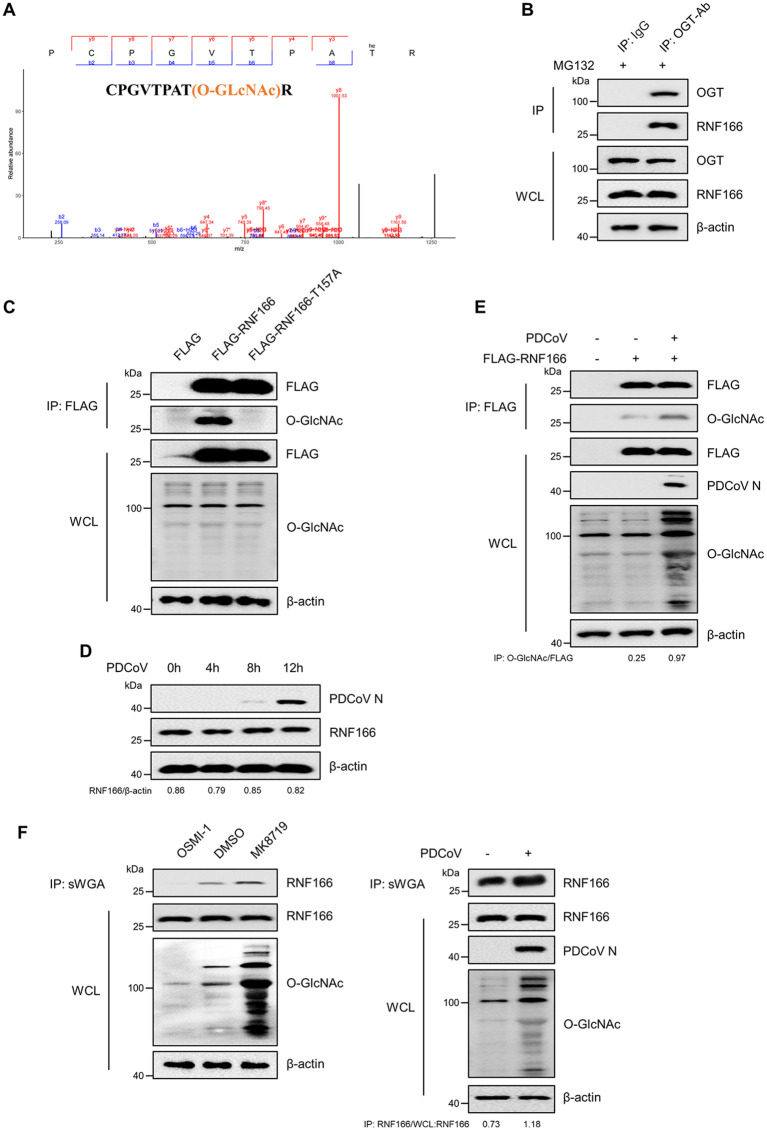
PDCoV infection increases O-GlcNAcylation of RNF166 at T157. **A**, Mapping of O-GlcNAcylation sites on RNF166 by mass spectrometry. **B**, Endogenous interaction between RNF166 and OGT in IPI-2I cells analyzed by co-immunoprecipitation (Co-IP) assay using an anti-OGT antibody, with normal IgG serving as control. Whole-cell lysate (WCL) and Co-IP complexes were immunoblotted with antibodies against OGT and RNF166. **C**, O-GlcNAcylation levels of wild-type RNF166 and its mutant RNF166-T157A in HEK293T cells transfected with FLAG-RNF166 or FLAG-RNF166-T157A, assessed by IP assay using an anti-FLAG antibody. WCL and Co-IP complexes were analyzed by western blot with antibodies against FLAG and O-GlcNAc. **D**, RNF166 protein expression in IPI-2I cells at the indicated time points after PDCoV infection by western blot. Ratios below the images represent the protein levels of RNF166 relative to β-actin, as quantified by the ImageJ software. **E**, IPI-2I cells expressing FLAG-RNF166, mock-infected or infected with PDCoV (MOI = 2), analyzed at 12 hpi for RNF166 O-GlcNAcylation by Co-IP assay with an anti-FLAG antibody. Ratios below the images represent the protein levels of IP (O-GlcNAc) relative to IP (FLAG), as quantified by the ImageJ software. **F**. O-GlcNAcylation of endogenous RNF166 assessed by succinylated wheat germ agglutinin (sWGA) pull-down assay in IPI-2I cells treated with MK-8719 (O-GlcNAcylation agonist), OSMI-1 (O-GlcNAcylation inhibitor), or infected with PDCoV. Ratios below the images represent the protein levels of IP (RNF166) relative to WCL (RNF166) as quantified by the ImageJ software.

Mass spectrometry analysis revealed that RNF166 (UniProt: A0A287A9W7) is O-GlcNAcylated specifically at the threonine residue T157 (Fig 3A). To validate this result, Co-IP assay was performed and the interaction between OGT and RNF166 was confirmed (Fig 3B), which is a prerequisite for the O-GlcNAcylation of RNF166. Subsequently, a eukaryotic expression plasmid encoding the RNF166 T157A mutant was constructed, which effectively blocks the O-GlcNAcylation of RNF166 at this site. Notably, we did not observe any O-GlcNAcylation signal in the RNF166-T157A mutant, suggesting that T157 may be the exclusive site of O-GlcNAcylation on RNF166 (Fig 3C). Additionally, as a restriction factor for PDCoV, the host did not increase the expression of RNF166 in response to PDCoV infection (Figs 3D and S2F). However, our results showed that the O-GlcNAcylation levels of RNF166 significantly increased following PDCoV infection, as determined by immunoprecipitation (IP) and succinylated wheat germ agglutinin (sWGA) pull-down assays (Fig 3E-3F). Therefore, O-GlcNAcylation at T157 may represent a crucial mechanism underlying the anti-PDCoV activity of RNF166.

### O-GlcNAcylation of RNF166 at T157 is critical for its antiviral function

**Fig 4:**
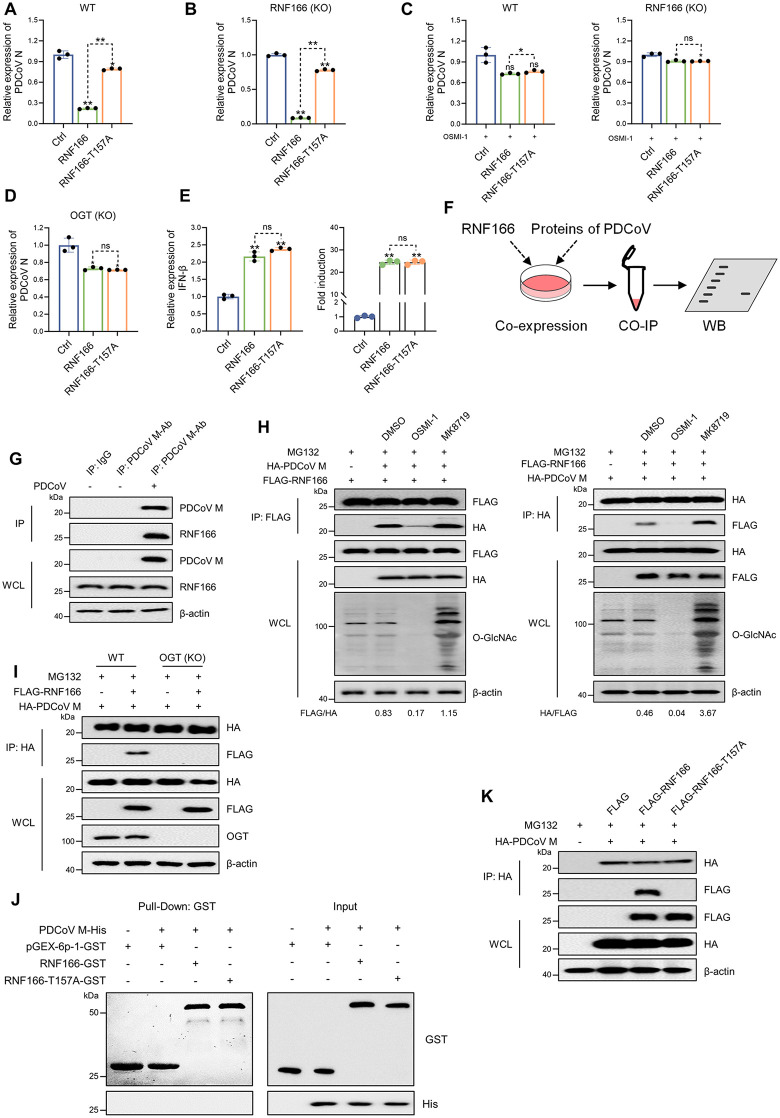
O-GlcNAcylation-dependent interaction between RNF166 and PDCoV M protein. **A-D**, PDCoV replication was assessed by RT-qPCR in the following cells expressing RNF166 or RNF166-T157A: (A) in wild-type IPI-2I cells. (B) in RNF166 (KO) IPI-2I cells. (C) in wild-type and RNF166 (KO) IPI-2I cells treated with OSMI-1 (50 μM). (D) in OGT (KO) IPI-2I cells. **E**, IFN-β mRNA levels and promoter activity in HEK293T cells transfected with RNF166 or RNF166-T157A, measured by RT-qPCR and dual-luciferase reporter assay. **F**, Schematic diagram of Identification of proteins encoded by PDCoV that interact with RNF166. **G**, Endogenous interaction between RNF166 and PDCoV M protein in PDCoV-infected IPI-2I cells, analyzed by Co-IP assay with anti-M and control IgG antibodies. **H**, Co-IP assay using anti-HA or anti-FLAG antibodies to analyze the RNF166–M interaction in HEK293T cells co-expressing HA-PDCoV M and FLAG-RNF166 in the presence of MG132 and treated with OSMI-1 (50 μM) or MK-8719 (10 μM). Ratios below the images represent the protein levels of FLAG relative to HA, or HA relative to FLAG as quantified by the ImageJ software. **I**, Interaction between RNF166 and PDCoV M protein in the presence of MG132, compared in wild-type versus OGT (KO) IPI-2I cells by Co-IP assay with an anti-HA antibody. **J**, Direct interaction between RNF166 and PDCoV M protein, assessed by GST pull-down assay using the purified GST-RNF166, GST-RNF166-T157A, and His-PDCoV M proteins expressed in *E. coli*. **K**, Co-IP assay using an anti-HA antibody to examine the interaction between RNF166-T157A and PDCoV M protein in HEK293T cells in the presence of MG132 co-transfected with HA-PDCoV M and FLAG-RNF166. Parallel experiments using FLAG-RNF166 as a control. Data in panels A–E represent mean ± s.d. (n = 3). Statistical significance was determined by two-tailed Student’s t-test; *P < 0.05; **P < 0.01; ns, not significant.

To assess the significance of RNF166 O-GlcNAcylation in its antiviral function, IPI-2I cells were transfected with either wild-type RNF166 or the T157A mutant, and viral replication was evaluated following PDCoV infection. The results showed that loss of O-GlcNAcylation at T157 markedly impaired the antiviral activity of RNF166 (Figs 4A and S4A). This phenotype was reproduced in RNF166 (KO) cells, thereby excluding interference from endogenous RNF166 (Figs 4B and S4B). Furthermore, treatment with OSMI-1, which inhibits intracellular O-GlcNAcylation, abolished the difference in anti-PDCoV activity between wild-type RNF166 and the T157A mutant (Figs 4C and S4C). Likewise, in OGT (KO) cells, where O-GlcNAcylation is completely absent, both wild-type RNF166 and the T157A mutant exhibited comparably weak antiviral activity (Figs 4D and S4D). Taken together, these findings indicate that O-GlcNAcylation at T157 of RNF166 is essential for its antiviral activity against PDCoV.

### RNF166 promotes interferon (IFN) production independently of O-GlcNAcylation

Previous studies have shown that RNF166 regulates intracellular IFN production through TRAF3 and TRAF6 in an indirect manner [35]. Coincidentally, PDCoV is an IFN-sensitive virus [36,37]. Thus, we aimed to ascertain whether the anti-PDCoV activity of RNF166 is linked to its regulatory role in IFN production. As shown in Fig 4E, RNF166 obviously increased IFN-β promoter activity and mRNA expression, as measured by dual-luciferase reporter assays and RT-qPCR. However, statistical analysis indicated no significant differences in IFN induction between RNF166 and the RNF166-T157A mutant. Likewise, no significant differences were observed in the expression of IFN-stimulated genes (ISGs) under both conditions (S4E Fig). Collectively, these results suggest that O-GlcNAcylation is not essential for the IFN-inducing activity of RNF166.

### O-GlcNAcylation enables interaction between RNF166 and the PDCoV M protein

As an E3 ubiquitin ligase, we hypothesized that RNF166 possesses the capability to directly degrade viral proteins through ubiquitination. To test this hypothesis, we co-expressed various PDCoV-encoded proteins with RNF166 in HEK293T cells and screened for viral proteins that interact with RNF166 using Co-IP assay (Fig 4F). Given that RNF166 is an E3 ubiquitin ligase, all samples were treated with MG132, a proteasome inhibitor, to prevent the degradation of ubiquitinated proteins. Our results showed that RNF166 interacts with the M protein of PDCoV (S5A-B Fig), and this finding was further corroborated by endogenous Co-IP assays in PDCoV-infected IPI-2I cells (Fig 4G).

Next, we examined the necessity of O-GlcNAcylation for the interaction between RNF166 and the PDCoV M protein. To this end, HEK293T cells were co-transfected with RNF166 and PDCoV M, followed by treatment with OSMI-1 or MK-8719 to modulate cellular O-GlcNAcylation levels. Co-IP assays revealed that reducing O-GlcNAcylation with OSMI-1 markedly diminished the RNF166–PDCoV M interaction compared to controls. Conversely, MK-8719 treatment displayed a trend toward enhanced interaction (Fig 4H), indicating that O-GlcNAcylation influences the binding between RNF166 and PDCoV M protein. Furthermore, we utilized OGT (KO) cells, which completely eliminate O-GlcNAcylation in vivo, as well as an *E. coli* expression system, which lacks O-GlcNAcylation in vitro, to express RNF166 and PDCoV M protein in both eukaryotic and prokaryotic systems, respectively. Co-IP and GST pull-down assays demonstrated that the interaction between RNF166 and PDCoV M protein was abolished in the absence of O-GlcNAcylation across both models (Fig 4I-4J). Additionally, RNF166-T157A, which lacks O-GlcNAcylation at T157, did not exhibit interaction with PDCoV M protein in exogenous Co-IP assays, compared to wild-type RNF166 (Fig 4K). Taken together, these results confirm that the interaction between RNF166 and PDCoV M protein is mediated by O-GlcNAcylation at T157 of RNF166, rather than by direct binding.

### O-GlcNAcylated RNF166 degrades PDCoV M protein via the ubiquitin-proteasome pathway

**Fig 5:**
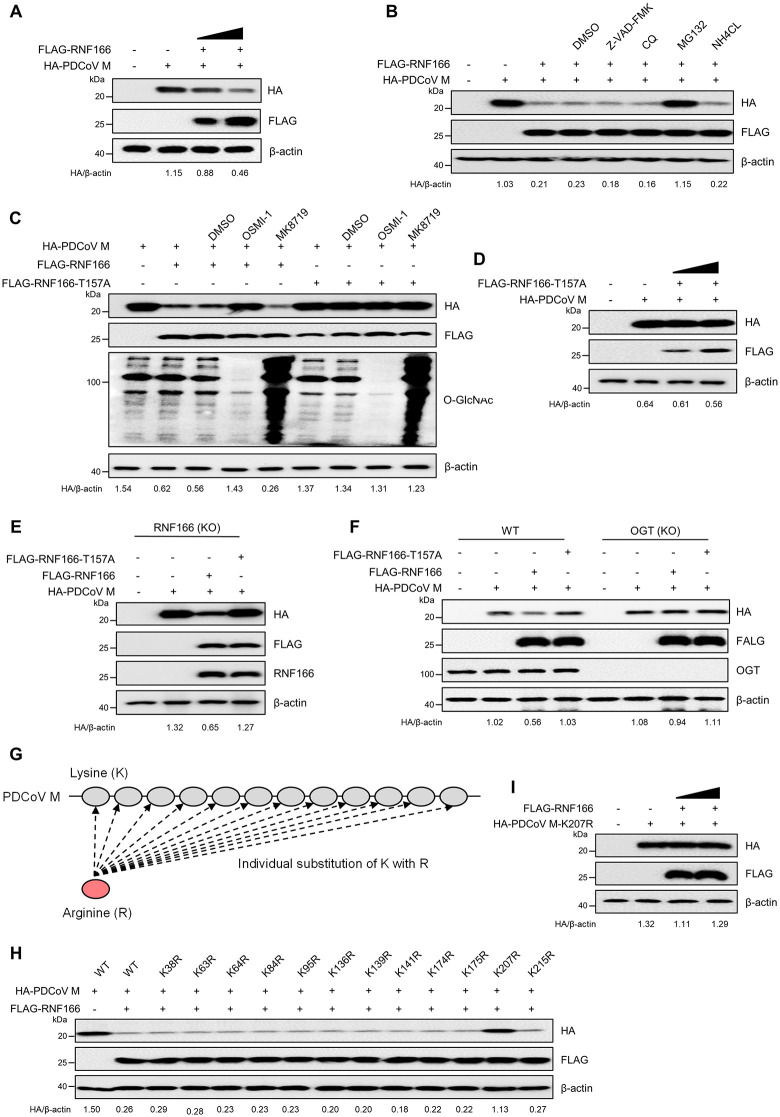
O-GlcNAcylated RNF166 mediates ubiquitination-dependent degradation of PDCoV M protein. **A**, Western blot to assess M protein degradation in HEK293T cells co-expressing HA-PDCoV M and increasing amounts of FLAG-RNF166. Ratios below the images represent the protein levels of HA relative to β-actin, as quantified by the ImageJ software. **B**, Effects of Z-VAD-FMK (apoptosis inhibitor), MG132 (proteasome inhibitor), NH_4_Cl, and CQ (chloroquine, autophagy inhibitor) on RNF166-mediated PDCoV M degradation. Ratios below the images represent the protein levels of HA relative to β-actin, as quantified by the ImageJ software. **C**, HEK293T cells co-expressing HA-PDCoV M with FLAG-RNF166 or the T157A mutant, treated with OSMI-1 (O-GlcNAcylation inhibitor) or MK-8719 (O-GlcNAcylation agonist) to evaluate their effects on M protein degradation. Ratios below the images represent the protein levels of HA relative to β-actin, as quantified by the ImageJ software. **D**, Western blot analysis of M protein expression in HEK293T cells co-transfecting with HA-PDCoV M and increasing amounts of FLAG-RNF166-T157A. Ratios below the images represent the protein levels of HA relative to β-actin, as quantified by the ImageJ software. **E–F,** Effects of RNF166-T157A on PDCoV M protein degradation in RNF166 (KO) IPI-2I cells **(E)** or OGT (KO) IPI-2I cells **(F)** co-transfected with FLAG-RNF166-T157A and HA-PDCoV M. Parallel experiments using FLAG-RNF166 as a control. Ratios below the images represent the protein levels of HA relative to β-actin, as quantified by the ImageJ software. **G,** Schematic diagram of representation of lysine (K) residue mutants in the PDCoV M protein. **H,** Identification of the lysine residues in PDCoV M protein targeted by RNF166 for degradation by co-transfecting FLAG-RNF166 and HA-PDCoV M or its lysine point mutants into HEK293T cells. Ratios below the images represent the protein levels of HA relative to β-actin, as quantified by the ImageJ software. **I,** Expression of PDCoV M mutant (M-K207R) in RNF166 (KO) IPI-2I cells co-transfecting with HA-PDCoV M-K207R and increasing amounts of FLAG-RNF166. Ratios below the images represent the protein levels of HA relative to β-actin, as quantified by the ImageJ software.

To investigate the biological consequences of the O-GlcNAcylation–mediated interaction between RNF166 and PDCoV M protein, we co-expressed the M protein with graded amounts of RNF166 in HEK293T cells. The results showed that increased expression of RNF166 led to a dose-dependent decrease in M protein levels (Fig 5A). Similarly, cycloheximide (CHX) chase assay further confirmed that RNF166 promoted the degradation of PDCoV M protein (S6A Fig). Subsequently, we examined the effects of pathway-specific inhibitors, including the proteasome inhibitor MG132, the lysosomal inhibitor NH_4_Cl, the autophagy inhibitor CQ, and the apoptosis inhibitor Z-VAD-FMK, to identify the pathway responsible for this reduction. As shown in Fig 5B, only MG132 effectively blocked the degradation of PDCoV M protein induced by RNF166. In contrast, CQ, NH_4_Cl, and Z-VAD-FMK did not exhibit any significant effects, suggesting that RNF166 targets the PDCoV M protein for proteasomal degradation.

Given that O-GlcNAcylation is essential for the interaction between RNF166 and the PDCoV M protein, we hypothesized that this modification is necessary for RNF166-mediated degradation of M protein. As expected, treatment with OSMI-1 to disrupt the RNF166–M interaction significantly suppressed RNF166-mediated degradation of the PDCoV M protein (Figs 4H-K and 5C). Consistently, OGA overexpression reduced RNF166 O-GlcNAcylation and also impaired the RNF166-driven degradation of the PDCoV M protein (S6B and S6C Fig). In addition, the O-GlcNAcylation-deficient mutant RNF166-T157A, which is unable to interact with the PDCoV M protein (Fig 4K), also failed to mediate its degradation (Fig 5D). These results were further validated in RNF166 (KO) cells (Fig 5E). Importantly, RNF166 no longer exhibited degradation activity toward the PDCoV M protein in the OGT (KO) cell line (Fig 5F). In summary, these findings suggest that O-GlcNAcylation is indispensable for RNF166-mediated degradation of the PDCoV M protein via the ubiquitin-proteasome pathway.

### K207 is the major ubiquitination degradation site of the M protein

Next, we aimed to identify the specific ubiquitination sites on the PDCoV M protein targeted by RNF166. Twelve mutants of the M protein were generated by substituting lysine (K) with arginine (R), and each mutant was co-expressed with RNF166 (Fig 5G). The results demonstrated that the K207R mutation in the M protein conferred resistance to RNF166-mediated degradation, even at elevated levels of RNF166 expression (Fig 5H-5I). Furthermore, this finding was corroborated in RNF166 (KO) cells (S7A Fig). Since RNF166 functions as an E3 ubiquitin ligase, we hypothesized that this resistance may be attributed to the absence of ubiquitination on the PDCoV M protein. Indeed, RNF166 enhanced the ubiquitination of the wild-type M protein, while it did not affect the M-K207R mutant (S7B Fig). Collectively, these results indicate that K207 is the key ubiquitination site necessary for RNF166-mediated degradation of the PDCoV M protein.

### Recombinant PDCoV with M-K207R mutation exhibits resistance to the antiviral effects of O-GlcNAcylated RNF166

**Fig 6:**
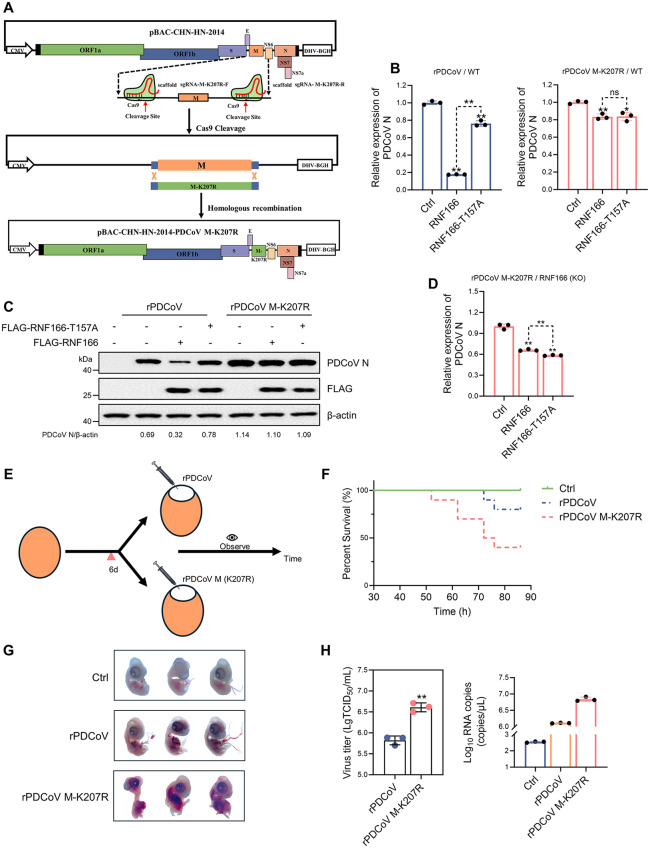
Recombinant PDCoV with M-K207R mutation resists the antiviral effects of O-GlcNAcylated RNF166. **A**, Schematic diagram of the construction of the infectious clone plasmid pBAC-CHN-HN-2014-M-K207R, with the rescued recombinant virus named rPDCoV M-K207R and the wild-type virus named rPDCoV. **B-C,** Comparison of the replication of rPDCoV and rPDCoV M-K207R in IPI-2I cells transfected with RNF166 or RNF166-T157A by RT-qPCR (**B**) and western blot **(C)**. **D**, Replication of rPDCoV M-K207R in wild-type and RNF166 (KO) IPI-2I cells. **E**, Schematic diagram of the chicken embryo infection experiment. Ratios below the images represent the protein levels of PDCoV N relative to β-actin, as quantified by the ImageJ software. **F**, Survival curves of chicken embryos infected with rPDCoV or rPDCoV M-K207R (3 × 10^5^ TCID_50_). G, Representative images of hemorrhagic lesions in the infected chicken embryos at 72 hpi. **H**, Viral RNA levels and titres in allantoic fluid from succumbed embryos. Data in panels B, D, and H represent mean ± s.d. (n = 3). Statistical significance was determined by two-tailed Student’s t-test; *P < 0.05; **P < 0.01; ns, not significant.

After identifying K207 on the PDCoV M protein as a site targeted by RNF166, we constructed a recombinant PDCoV carrying the M-K207R mutation, designated rPDCoV M-K207R, using the infectious clone of PDCoV strain CHN-HN-2014 (Figs 6A and S8A). This mutant was anticipated to resist O-GlcNAcylated RNF166–mediated ubiquitin-proteasome degradation of the M protein, with wild-type PDCoV (rPDCoV) rescued in parallel as a control. Subsequently, IPI-2I cells were transfected with either wild-type RNF166 or the T157A mutant, and viral replication was evaluated following infection with rPDCoV or rPDCoV M-K207R. As expected, RNF166-T157A exhibited significantly weaker antiviral capabilities than wild-type RNF166 when infected with rPDCoV (Fig 6B-6C). However, infection with rPDCoV M-K207R markedly impaired the antiviral activity of RNF166, and no differences in antiviral capabilities were observed between wild-type RNF166 and RNF166-T157A (Figs 6B-6C and S8B). This observation was further corroborated in RNF166 (KO) cells (Figs 6D and S8C), indicating that K207 of the M protein is essential for the antiviral activity of O-GlcNAcylated RNF166.

Chicken embryos have been established as an effective model for PDCoV infection in vivo [38]. We further investigated whether the absence of RNF166-mediated M protein degradation in rPDCoV-M-K207R leads to increased pathogenicity in vivo. As expected, following infection of chicken embryos with equivalent doses of the viruses, rPDCoV-M-K207R caused more severe hemorrhaging, higher viral loads, and increased mortality compared to rPDCoV (Fig 6E-6H). Collectively, these data underscore the biological role of O-GlcNAcylation at T157 of RNF166, which functions to degrade the PDCoV M protein at the K207 site through the ubiquitin-proteasome pathway, thereby inhibiting viral replication.

### HBP‑driven O‑GlcNAcylation of RNF166 degrades M proteins of porcine but not human or murine coronaviruses

**Fig 7:**
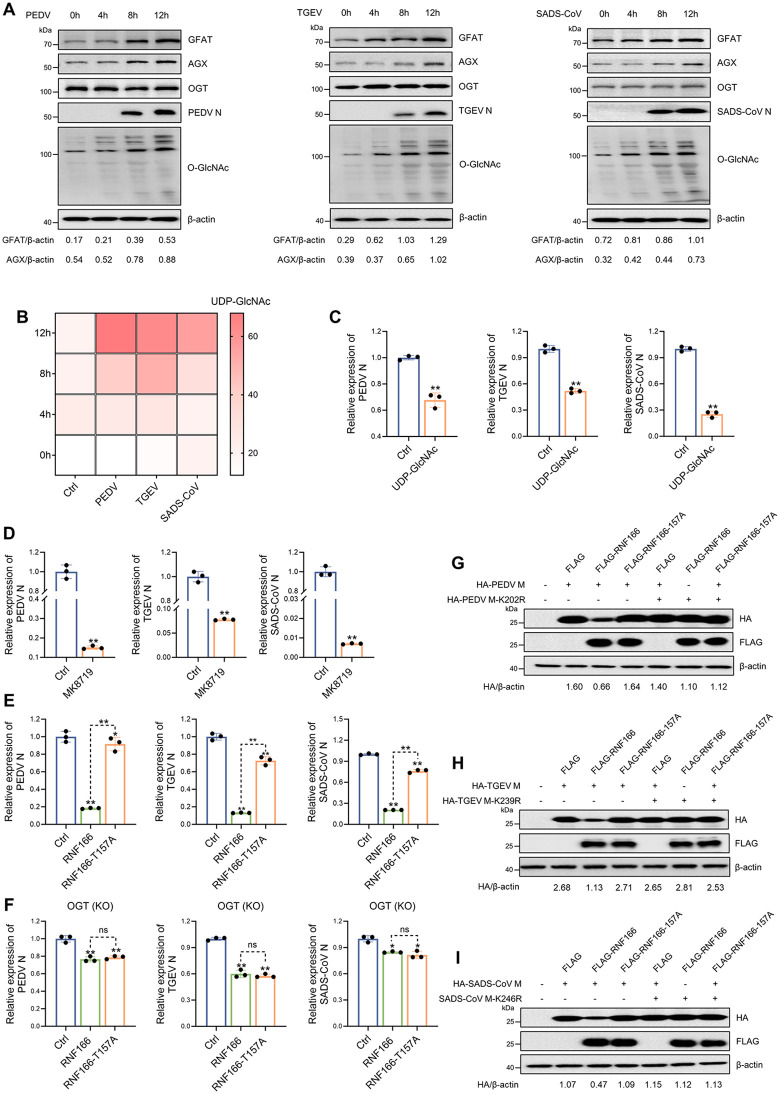
HBP‑driven O‑GlcNAcylation of RNF166 degrades M proteins of porcine but not human or murine coronaviruses. **A-B,** HBP flux in IPI-2I cells infected with PEDV, TGEV, or SADS-CoV at indicated time points: **(A)** Protein levels of GFAT, AGX, OGT, viral N protein, and global O-GlcNAcylation, assessed by western blot. Ratios below the images represent the protein levels of GFAT and AGX relative to β-actin, as quantified by the ImageJ software. **(B)** Intracellular UDP-GlcNAc content, measured by ELISA. **C,** Viral replication of PEDV, TGEV, or SADS-CoV in IPI-2I cells supplemented with or without UDP-GlcNAc. **D**, Viral replication of PEDV, TGEV, or SADS-CoV in IPI-2I cells treated with or without the O-GlcNAcylation agonist MK-8719. **E-F**, Viral replication of PEDV, TGEV, or SADS-CoV in wild-type (**E**) and RNF166 (KO) **(F)** IPI-2I cells overexpressing RNF166 or RNF166-T157A. **G-I**, Western blot analysis of the degradation of PEDV M protein **(G)**, TGEV M protein **(H)**, SADS-CoV M protein (**I**) and their corresponding mutants (PEDV M-K202R, TGEV M-K239R, SADS-CoV M-K246R) in HEK293T cells transfected with FLAG-RNF166 or FLAG-RNF166-T157A. Ratios below the images represent the protein levels of HA relative to β-actin, as quantified by the ImageJ software. Data in panels B–F represent mean ± s.d. (n = 3). Statistical significance was determined by two-tailed Student’s t-test; *P < 0.05; **P < 0.01; ns, not significant.

Using PDCoV as a model, we have demonstrated that HBP links metabolism and innate immunity through O-GlcNAcylation, with the HBP end product UDP-GlcNAc being transferred by OGT to RNF166 at T157. This modification licenses RNF166 to degrade the PDCoV M protein. We next examined whether this mechanism is operational in other porcine coronaviruses, including porcine epidemic diarrhea virus (PEDV), transmissible gastroenteritis virus (TGEV) and swine acute diarrhea syndrome coronavirus (SADS-CoV). Porcine respiratory coronavirus (PRCV) and porcine hemagglutinating encephalomyelitis virus (PHEV) were not included in our analysis due to the limited availability of reagents. All three viruses (PEDV, TGEV, and SADS-CoV) triggered enhanced HBP flux (Figs 7A-7B and S9A-C), and supplementation with either UDP-GlcNAc or the O-GlcNAcase inhibitor MK-8719 suppressed replication in each virus (Figs 7C-7D and S9D-F), indicating that O-GlcNAcylation contributes to a common host antiviral defense in these infections. Similarly, RNF166 and its O-GlcNAcylation-deficient mutant T157A exhibited differential antiviral activity against each virus (Fig 7E). This difference was abolished by OGT deficiency (Fig 7F), consistent with RNF166 possessing both O-GlcNAcylation-dependent and O-GlcNAcylation-independent functions. In vitro, RNF166 degraded the M proteins of PEDV, TGEV, and SADS-CoV, while the RNF166-T157A mutant did not, demonstrating that O-GlcNAcylation is essential for its E3 ubiquitin ligase activity. Sequence alignment revealed that the lysine residue K207 in the PDCoV M protein is highly conserved at equivalent positions in the M proteins of other porcine coronaviruses: K202 in PEDV, K206 in PHEV, K238 in PRCV, K239 in TGEV, and K246 in SADS-CoV (S10A Fig).

To assess whether the conserved lysine residues are likewise required for RNF166-mediated degradation, each lysine residue was substituted with arginine (PEDV M-K202R, TGEV M-K239R and SADS-CoV M-K246R). These substitutions resulted in the complete abolition of RNF166-mediated degradation of the corresponding M proteins (Fig 7G-7I), indicating that RNF166 targets a conserved lysine across porcine coronaviruses and that variations at this position confer resistance to degradation. Notably, T157 O-GlcNAcylation enables RNF166 to degrade M proteins exclusively within porcine coronaviruses, while this activity is absent against the M proteins of human coronaviruses, including HCoV-229E and SARS-CoV-2, as well as the murine coronavirus MHV (S10B-D Fig). Consistently, the lysine residue corresponding to PDCoV K207 is not conserved in HKU11, HCoV-229E, HCoV-NL63, HCoV-OC43, MHV, HCoV-HKU1, MERS-CoV, SARS-CoV, SARS-CoV-2, IBV, or TCoV (S10E Fig). Therefore, RNF166-mediated degradation of M proteins occurs in a host-restricted manner, specifically targeting porcine coronaviruses.

## Discussion

Certain carbohydrates, such as heparan sulfate and chitosan, exhibit potent inhibitory effects against a range of viruses, including HIV, influenza virus, and herpesviruses [39–41]. Clinically, the oral administration of D-glucosamine has been employed to decrease viral load in patients with SARS-CoV-2 [42]. Consequently, the use of natural metabolites to inhibit viral replication presents promising therapeutic prospects. In this study, we demonstrate that UDP-GlcNAc exerts significant antiviral effects through O-GlcNAcylation. Unlike conventional targeted antivirals, UDP-GlcNAc exerts its antiviral effects by enhancing the host’s immune responses, offering unique advantages both in prophylactic treatment and in countering rapid viral mutations. Furthermore, when treatments are directed at animals, particularly those entering the human food chain, UDP-GlcNAc mitigates the risk of drug residues in animals, thereby reducing the potential for biomagnification [43].

The RING finger protein family is widely recognized as an effective regulator of antiviral immunity [44,45]. For example, RNF144A enhances the cGAS–cGAMP–STING signaling pathway against DNA viruses by promoting the translocation of STING [46]. RNF81 responds to RNA virus infections by stabilizing MAVS, which triggers the activation of IRF3 and NF-κB [47,48]. In this study, we demonstrate that RNF166 inhibits porcine coronavirus replication through two distinct mechanisms. The first mechanism involves the independent upregulation of IFN expression, while the second mechanism relies on O-GlcNAcylation at the T157 site, which promotes the degradation of the viral M protein. The latter mechanism represents the primary pathway for inhibiting viral replication, thereby expanding our understanding of the antiviral functions of the RNF family. Moreover, recent studies have shown that SARS-CoV-2 can directly degrade RIG-I through the proteasomal pathway by exploiting RNF135 [49]. Together, these observations suggest that the reciprocal strategies by which hosts deploy, and viruses exploit, RNF proteins represent only a fraction of an intricate molecular arms race that is yet to be fully elucidated.

An intriguing finding from our study is that RNF166 selectively degrades the M proteins of porcine coronaviruses in a host-restricted manner, while showing no activity toward the M proteins of other coronaviruses, such as HCoV-229E and SARS-CoV-2 (S10B-D Fig). We propose four non-mutually exclusive explanations for this species-specific activity. First, the absence of a conserved ubiquitination site in the M protein may hinder effective targeting by RNF166. For example, the lysine residue K207 in the PDCoV M protein is highly conserved among porcine coronaviruses but is absent in other coronaviruses, including SARS-CoV-2, SARS-CoV, MERS-CoV, HCoV-229E, MHV, and HCoV-OC43 (S10E Fig). Second, even when a similar lysine residue is present, its ubiquitination may still depend on whether the site is structurally exposed [50–52]. Differences in membrane topology and conformation among coronavirus M proteins may reduce the accessibility of this residue to RNF166, thereby limiting RNF166-mediated ubiquitination [53,54]. Third, variations in the subcellular localization of M proteins among different coronaviruses may alter the likelihood of physical encounters with RNF166, thereby reducing the efficiency of ubiquitination [55–57]. Fourth, the tissue distribution of RNF166 may further shape the biological relevance of its selective antiviral activity in vivo. Public expression data indicate that RNF166 is widely expressed across various tissues, with relatively higher expression levels in the spleen, lymph nodes, and intestines [58]. This expression pattern may be compatible with the enteric tropism of porcine coronaviruses. Consistent with this, RNF166 in our study mediated the degradation of M proteins from several porcine enteric coronaviruses, including PDCoV, PEDV, TGEV, and SADS-CoV. Collectively, these differences in sequence, structure, spatial distribution and tissue localization may elucidate why RNF166 degrades M proteins from porcine coronaviruses but not from other coronavirus species.

PDCoV M K207 serves not only as a site for ubiquitination but also as an acetylation site [59]. Research has shown that after SIRT5-mediated desuccinylation of the M protein at K207, the M protein is relocalized to peroxisomes, which act as antiviral signaling hubs [60,61]. Within these organelles, the M protein promotes peroxisomal degradation via autophagy, thereby broadly undermining the immune defenses and enhancing viral replication [59]. However, this apparent advantage may be transient. In the absence of succinylation, the M protein may adopt a quiescent conformation, rendering it susceptible to rapid elimination through RNF166-mediated ubiquitination. This sequence of events suggests that the host may convert a viral advantage into a liability through precise post-translational modifications.

O‑GlcNAcylation has been extensively characterized as a key driver of tumorigenesis [62,63,64]. However, its role in antiviral immunity remains controversial, with evidence supporting both protective and promotive effects on viral infection [11]. This paradox may stem from the complex interplay between pathogens and host immune responses [65,66]. In addition, progress in this field has been hampered by technical challenges [67,68]. The highly dynamic and reversible nature of O‑GlcNAcylation, coupled with its low stoichiometry, the absence of a reliable sequence motif, and the requirement for specialized enrichment combined with high‑sensitivity mass spectrometry, make reliable site‑specific identification particularly challenging [69,70]. Consequently, relatively few modification sites have been mapped with sufficient confidence to permit functional studies [68,69]. In this study, we provide a dataset of 105 O‑GlcNAcylated proteins identified during PDCoV infection. This dataset greatly enriches the current O‑GlcNAcylation database and will strongly promote future mechanistic and functional studies. Notably, despite the PDCoV spike (S) glycoprotein being heavily glycosylated, no O‑GlcNAcylation sites were detected in our dataset [71,72]. This may reflect intrinsically low modification levels or a preference for alternative glycosylation types such as N‑glycosylation, O‑fucosylation, O‑xylosylation or O‑mannosylation.

**Fig 8:**
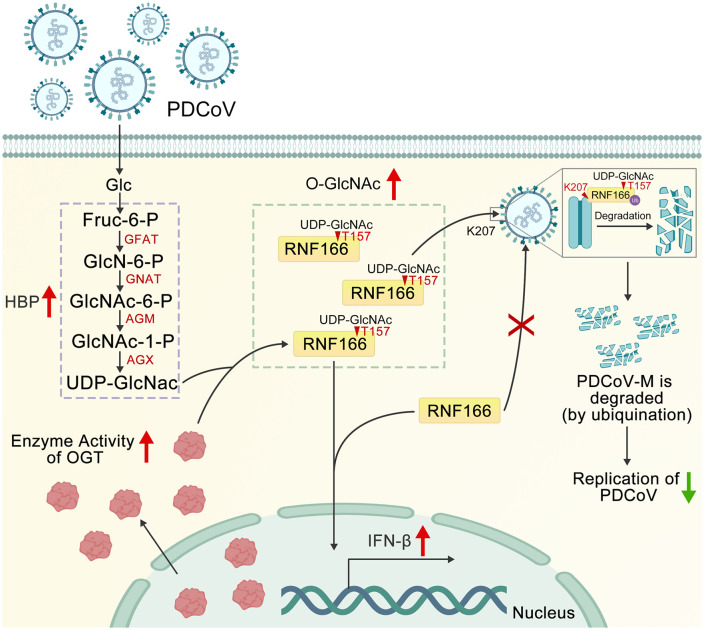
Proposed model for the mechanism by which O-GlcNAcylated RNF166 degrades the M protein of porcine coronaviruses. Using PDCoV as a representative example, in response to porcine coronavirus infection, host cells enhance the HBP flux, leading to UDP-GlcNAc accumulation. Concurrently, OGT translocates to the cytoplasm and catalyzes the O-GlcNAcylation of RNF166 at T157. This modification licenses RNF166 to mediate the degradation of the PDCoV M protein, while it does not alter the ability of RNF166 to induce interferon production. This mechanism is highly conserved across porcine enteric coronaviruses.

Overall, our findings demonstrate that UDP-GlcNAc drives metabolic intervention through O-GlcNAcylation, which licenses RNF166 to selectively degrade porcine coronavirus M proteins (Fig 8), revealing a coordinated axis of metabolism, ubiquitination, and O-GlcNAcylation in antiviral defense.

## Materials and methods

### Cells, viruses and reagents

IPI-2I cells, Vero cells, and HEK293T cells were cultured in Dulbecco’s modified Eagle’s medium (DMEM) (Invitrogen, NY, USA) supplemented with 10% heat-inactivated fetal bovine serum (Vazyme, Nanjing, China) at 37°C in 5% CO_2_. PDCoV strain CHN-HN-2014 (GenBank accession number KT336560), PEDV strain AJ1102 (GenBank accession number JX188454.1), TGEV strain WH1 (GenBank accession number HQ462571), and SADS-CoV strain CHN-GD-2017 (GenBank accession number MH539766) were isolated and stored in our laboratory [73]. Antibodies against AGX (A8662), GFAT1 (A20873), β-actin (AC050), and RNF166 (A8276) were purchased from ABclonal, China. Antibody against O-GlcNAc (9875) was purchased from Cell Signaling Technology, USA; antibody against OGT (66823–1) was purchased from ProteinTech, USA; antibodies against HA (M180-3) and FLAG (M185-3) were purchased from Medical & Biological Laboratories, Japan. Antibodies against PDCoV N, PDCoV M, PEDV N, and TGEV N were generated in-house. The antibody against SADS-CoV N was kindly provided by Dr. Hao Zhang at Sun Yat-sen University. UDP-GlcNAc (HY-112174) was purchased from MedChemExpress, China. OSMI-1 (S9835), Cycloheximide (S7418) and MK-8719 (S8890) were obtained from Selleck Chemicals, USA. Cas9 Nuclease (M0386S) and PNGase F (P0704S) were acquired from New England Biolabs, USA, and sWGA lectin (AL-1023S) was purchased from Vector Laboratories, USA.

### Plasmid construction and transfection

The full-length cDNA of porcine RNF166, OGT, OGA, PDCoV M, PEDV M, TGEV M, SADS-CoV M, HCoV-229E M, MHV M, and SARS-CoV-2 M was amplified by PCR and subcloned into pCAGGS-FLAG/HA, pGEX-6P-1, or pET28a expression vectors. Site-directed mutants, including OGT (H558E, Y841A), RNF166 (T157A), PDCoV-M (K38R, K63R, K64R, K84R, K95R, K136R, K139R, K141R, K174R, K175R, K207R, K207E, and K215R), PEDV M (K202R), TGEV M (K239R) and SADS-CoV M (K246R), were generated using overlap extension PCR and inserted into the pCAGGS-FLAG/HA vector. The plasmids pIFN β-Luc, pIFN β-TK, pBAC-CHN-HN-2014, and PDCoV-HA constructs encoding nsp3–nsp16, S, E, M, N, NS6, NS7, and NS7a were maintained in our laboratory. Primer sequences used for all cloning procedures are listed in S1 Table. Plasmid transfections were performed with jet PRIME (101000046, Polyplus, France) according to the manufacturer’s instructions. All primer information is provided in S1-S4 Table.

### UDP-GlcNAc and OGT enzymatic activity assay

Intracellular UDP-GlcNAc levels and OGT activity were quantified using ELISA kits (MM-78502O1 and MM-78245O1, MEIMIAN, China) according to the manufacturer’s instructions. Briefly, cells were lysed by sonication on ice and clarified by centrifugation at 12,000 × g for 10 min. The supernatants were then applied to 96-well plates pre-coated with specific antibodies and incubated at 37 °C. After incubation with HRP-conjugated secondary antibodies, the reactions were developed with TMB substrate and subsequently stopped; the absorbance was recorded at 450 nm.

### Global profiling of O-GlcNAcylation–modified proteins

IPI-2I cells were infected with PDCoV (MOI = 2) for 12 h. Cells were then harvested and sent to Jingjie BioTech (Hangzhou, China) for proteomic analysis. Protein extraction, tryptic digestion, enrichment of O-GlcNAc–modified peptides, and LC–MS/MS were performed according to standard protocols. Analysis of the subcellular localization of the proteins was performed based on data from the Human Protein Atlas database (https://www.proteinatlas.org/). Information on experimentally identified O-GlcNAcylated proteins and modification sites was obtained from O-GlcNAcAtlas (https://oglcnac.org).

### Nuclear and cytoplasmic fractionation

Nuclear and cytoplasmic proteins were separated using the Beyotime Nuclear and Cytoplasmic Protein Extraction Kit (catalog no. P0027, Beyotime, China) according to the manufacturer’s instructions. Cells were collected and resuspended in ice-cold cytoplasmic protein extraction reagent A, vortexed vigorously, and incubated on ice for 10 min. Extraction reagent B was then added, and samples were vortexed at high speed for 5 s. The lysates were centrifuged at 12,000 × g for 5 min at 4 °C to pellet the nuclei and release the cytoplasmic proteins into the supernatant. The supernatant (cytoplasmic fraction) was transferred to a fresh tube and kept on ice. The nuclear pellet was washed once with reagent A, resuspended in nuclear protein extraction reagent, alternately vortexed and incubated on ice for 10 min, and then centrifuged at 12,000 × g for 5 min at 4 °C. The resulting supernatant (nuclear fraction) was collected for downstream analysis. Protein concentrations in both fractions were determined by BCA assay, and equal amounts of protein were analyzed by SDS–PAGE and immunoblotting with the indicated antibodies.

### Immunoprecipitation

Immunoprecipitation was performed using the Thermo Scientific Pierce Direct IP Kit (catalog no. 26148, Thermo Fisher Scientific, USA) according to the manufacturer’s instructions. Cells overexpressing the indicated proteins, or infected with virus, or in a resting (unstimulated) state were lysed in the kit-supplied buffer containing protease and phosphatase inhibitors. The lysates were then clarified by centrifugation at 12,000 × g for 10 min at 4 °C. Two micrograms of antibody were covalently coupled to the AminoLink Plus Coupling Resin and incubated with 500 µg of clarified lysate for 2 h at 4 °C with gentle rotation. After three washes with the kit wash buffer, the bound proteins were eluted with the kit elution buffer and analyzed by SDS–PAGE and immunoblotting using the indicated antibodies.

### Co-immunoprecipitation

Co-immunoprecipitation was carried out with the Thermo Scientific Pierce Co-IP Kit (catalog no. 26149, Thermo Fisher Scientific, USA) according to the manufacturer’s instructions. Briefly, cells overexpressing the indicated proteins, or infected with virus, or in a resting (unstimulated) state were lysed in the kit-supplied buffer containing protease and phosphatase inhibitors. The lysates were clarified by centrifugation at 12,000 × g for 10 min at 4 °C. Two micrograms of antibody were immobilized on AminoLink Plus Coupling Resin and incubated with 500 µg of clarified lysate for 2 h at 4 °C with gentle rotation. After three washes with the kit wash buffer, the immune complexes were eluted with the kit elution buffer and analyzed by SDS–PAGE and immunoblotting using the indicated antibodies.

### sWGA pull-down assay

Cell lysates were prepared in buffer containing 125 mM NaCl, 50 mM Tris-HCl (pH 7.4), 5 mM EDTA, 0.1% NP-40 and protease/phosphatase inhibitors. The lysates were then centrifuged at 12 000 × g for 10 min at 4 °C to remove debris, followed by a second centrifugation under identical conditions. The supernatants were denatured at 95 °C for 10 min in glycoprotein denaturing buffer, cooled to room temperature, and treated with PNGase F (P0704S, New England Biolabs, USA) according to the manufacturer’s instructions to remove N-linked glycans. After clarification at 12,000 × g for 10 min, samples were incubated with sWGA-conjugated agarose beads (AL-1023S, Vector Laboratories, USA) overnight at 4 °C with gentle rotation. The beads were washed three times with binding buffer (10 mM Tris-HCl pH 7.6, 150 mM NaCl, 1 mM CaCl_2_, 1 mM MnCl_2_), and bound glycoproteins were eluted by incubating with 0.5 M N-acetyl-D-glucosamine in binding buffer for 15 min at room temperature. The eluates were resolved by SDS–PAGE and analyzed by immunoblotting with the indicated antibodies. Input samples were normalized to total protein content prior to pull-down.

### GST pull-down assay

GST pull-down assays were performed using the Pierce GST Protein Interaction Pull-Down Kit (catalog no. 21516, Thermo Fisher Scientific, USA) according to the manufacturer’s instructions. Briefly, GST-tagged bait proteins were expressed in Escherichia coli BL21(DE3) upon induction with 0.5 mM IPTG at 16 °C for 16 h. The bacterial pellets were lysed in the kit-provided lysis buffer supplemented with protease inhibitors and clarified by centrifugation at 12,000 × g for 10 min at 4 °C. The supernatants containing GST fusions were incubated with glutathione resin for 1 h at 4 °C with gentle rotation to immobilize the bait proteins. After three washes with washing buffer, prey proteins (500 µg lysate) were added and incubated for 2 h at 4 °C. Unbound proteins were removed by five successive washes, and bound complexes were eluted by boiling in SDS sample buffer. The eluates were resolved by SDS–PAGE and analyzed by immunoblotting with the indicated antibodies.

### Establishment of cell lines by CRISPR–Cas9

Guide RNAs targeting the gene of interest were designed and cloned into the PX458 vector. PX458 plasmids were transfected into target cells using jet PRIME according to the manufacturer’s instructions. Twelve hours post-transfection, cells were harvested and single GFP-positive cells were sorted into 96-well plates by flow cytometry. Expanded clones were screened by genomic DNA extraction (K-101 kit, YSY BIOTECH, China) according to the manufacturer’s instructions, followed by PCR amplification and Sanger sequencing to confirm the formation of indels. The knockout efficiency was further validated by immunoblotting. The sequencing primers and gRNA target sequences are listed in S3 Table.

### Generation of recombinant virus

Recombinant PDCoV carrying the M-K207R substitution was generated by CRISPR/Cas9–mediated mutagenesis of the pBAC-CHN-HN-2014 bacterial artificial chromosome (BAC) infectious clone. Guide RNAs targeting the upstream (sgPDCoV-Ma) and downstream (sgPDCoV-Mb) regions of the M gene were synthesized in vitro. The pBAC-CHN-HN-2014 BAC was linearized by cleavage with Cas9 in the presence of sgRNA-Ma and sgRNA-Mb. A repair fragment encoding the K207R mutation was amplified by overlap extension PCR (primers listed in S3 Table) and introduced into the linearized BAC by homologous recombination, yielding pBAC-CHN-HN-2014-M-K207R. For virus rescue, 5 µg of recombinant BAC DNA was transfected into confluent LLC-PK1 cells in six-well plates using jet PRIME (101000046, Polyplus, France) according to the manufacturer’s instructions. Four hours post-transfection, cells were washed twice with PBS and overlaid with MEM supplemented with 7.5 µg/mL trypsin (Sigma-Aldrich, USA). Cultures were maintained at 37 °C and 5% CO_2_, with medium refreshed every two days. The cytopathic effect was monitored, and supernatants were harvested when > 75% CPE was observed. Viral stocks were clarified by low-speed centrifugation and stored at –80 °C.

### Pathogenicity assessment of recombinant virus

Pathogenicity was evaluated in 6-day-old specific-pathogen-free embryonated chicken eggs. In the virus-infected group, each egg was inoculated via the yolk sac with 3 × 10^5^ TCID_50_ of PDCoV in a total volume of 100 µL. The control group received 100 µL of culture medium inoculated via the same route. The inoculated eggs were incubated at 37 °C with constant humidity. Embryo viability was assessed daily by candling; death was confirmed by cessation of movement, ruptured yolk-sac vessels, evidence of disseminated intravascular coagulation, and extensive autolysis. Upon embryo death or at the end of the incubation period, allantoic fluid was harvested, clarified by low-speed centrifugation, and used to determine viral titer by TCID_50_ assay and viral RNA copy number by quantitative RT-qPCR.

### Statistical analysis

The experiments were independently repeated using the specified biological replicates as indicated in the figure legends. Statistical analyses were conducted using Prism 10 (https://www.graphpad.com). Western blot band intensities were quantified using ImageJ (https://imagej.nih.gov). The statistical methods are indicated in the corresponding figure legends. Two-tailed Student’s t-tests were applied to analyze data from two unpaired groups. Differences were considered to be statistically significant when the corresponding P values were less than 0.05.

## Supporting information

S1 Fig O-GlcNAcylation functions as a host restriction factor for PDCoV. **A**, Relative mRNA levels of GFAT and AGX, assessed by RT-qPCR. **B**, Cell viability was assessed by CCK-8 assay in IPI-2I cells supplemented with various concentrations of UDP-GlcNAc (mM). **C-I**, IPI‑2I cells were subjected to the indicated treatments, infected with PDCoV (MOI = 2), and harvested at 12 h post-infection (hpi) for viral replication analysis. (**C)** assessed by TCID50 assay, cells supplemented with or without UDP‑GlcNAc (32 mM). (**D-E)** Cells treated with the O‑GlcNAcylation agonist MK‑8719 or inhibitor OSMI‑1, assessed by TCID_50_ assay (D) and western blot (E). (**F)** Cells were transfected to overexpress OGA. (**G)** Wild-type versus OGT knockout (KO) IPI-2I cells, assessed by TCID_50_ assay. (**H-I)** OGT (KO) IPI-2I cells overexpressing OGT or catalytically inactive OGT‑DP, assessed by TCID_50_ assay (H) and western blot (I). Data in panels A, B, C, D, F, G and H represent mean ± s.d. (n = 3). Statistical significance was determined by two-tailed Student’s t-test; *P < 0.05; **P < 0.01; ns, not significant.
(TIF)

S2 Fig RNF166 inhibits PDCoV replication in cells. **A,** Cellular O-GlcNAcylation was assessed by western blot in IPI-2I cells at 12 hpi with various multiplicities of infection (MOIs). **B,** Grayscale intensity of OGT bands in the immunoblot shown in Fig 2D was quantified using ImageJ. **C,** Effect of RNF166 on PDCoV replication, as assessed in RNF166-overexpressing cells by western blot. **D-E,** Comparison of PDCoV replication between wild-type and RNF166 (KO) IPI-2I cells, assessed by TCID_50_ (D) assay and western blot (E). **F,** IPI-2I cells were infected with PDCoV (MOI = 2) and harvested at the indicated time points, followed by RT-qPCR analysis of RNF166 mRNA levels. Data in panels D and F represent mean ± s.d. (n = 3). Statistical significance was determined by two-tailed Student’s t-test; *P < 0.05; **P < 0.01; ns, not significant.
(TIF)

S3 Fig Functional enrichment analysis of O-GlcNAcylation targets following PDCoV infection. **A–C,** Identification of O-GlcNAcylated proteins by mass spectrometry from IPI-2I cells infected with PDCoV (MOI = 2) and harvested at 12 hpi, followed by enrichment analyses. (**A)** Gene Ontology (GO) enrichment. (**B)** Kyoto Encyclopedia of Genes and Genomes (KEGG) pathway. (**C)** Clusters of Orthologous Groups/Eukaryotic Orthologous Groups (COG/KOG).
(TIF)

S4 Fig Thr157 O-GlcNAcylation is essential for the antiviral activity of RNF166 but not for the induction of interferon-stimulated genes. **A-D**, PDCoV replication was assessed by TCID_50_ assay in the following cells expressing either RNF166 or the mutant RNF166-T157A: (**A**) in wild-type IPI-2I cells. (**B**) in RNF166 (KO) IPI-2I cells. (**C**) in wild-type and RNF166 (KO) IPI-2I cells treated with OSMI-1 (50 μM). (**D**) in OGT (KO) IPI-2I cells. **E,** The mRNA levels of ISG15, ISG54 and ISG56 in HEK293T cells transfected with RNF166 or RNF166-T157A, assessed by RT-qPCR. Data in panels A–E represent mean ± s.d. (n = 3). Statistical significance was determined by two-tailed Student’s t-test; *P < 0.05; **P < 0.01; ns, not significant.
(TIF)

S5 Fig Identification of proteins encoded by PDCoV that interact with RNF166. **A,** Screening for RNF166 partners that interact with PDCoV-encoded proteins in HEK293T cells, which were co-transfected with FLAG-RNF166 and HA-tagged PDCoV-encoded protein expression plasmids. Co-immunoprecipitation (Co-IP) was performed using an anti-FLAG antibody. Whole-cell lysates (WCL) and Co-IP complexes were immunoblotted with antibodies against HA and FLAG. **B,** IPI-2I cells were co-transfected with HA-tagged PDCoV M and FLAG-tagged RNF166, followed by indirect immunofluorescence analysis to assess their subcellular co-localization. PDCoV M is shown in green, RNF166 in red. Scale bar, 20 μm.
(TIF)

S6 Fig RNF166 mediates the degradation of the PDCoV M protein. **A,** HEK293T cells were co-transfected with RNF166 and PDCoV M, followed by CHX treatment and PDCoV M protein levels were analyzed by western blot. **B,** HEK293T cells were co-transfected with MYC-OGA, FLAG-RNF166, and HA-PDCoV M, followed by western blot analysis of the effect of OGA on RNF166-mediated PDCoV M degradation. **C,** O-GlcNAcylation levels of RNF166 in HEK293T cells transfected with FLAG-RNF166 in the presence or absence of MYC-OGA, assessed by IP assay using an anti-FLAG antibody. WCL and FLAG immunoprecipitates were analyzed by western blot with antibodies against FLAG, MYC, O-GlcNAc, and β-actin. Western blot band intensities were quantified using ImageJ.
(TIF)

S7 Fig RNF166 enhances the ubiquitination of PDCoV M protein at K207. **A,** Effects of RNF166 on PDCoV M-K207R protein degradation in RNF166 (KO) IPI-2I cells co-transfected with FLAG-RNF166 and HA-PDCoV M-K207R. Parallel experiments using HA-PDCoV M as a control. **B**, Ubiquitination levels of wild-type HA-PDCoV M and its mutant HA-PDCoV M-K207R in HEK293T cells co-transfected with FLAG-RNF166, HA-PDCoV M or FLAG-RNF166, HA-PDCoV M-K207R, assessed by Co-IP assay using an anti-HA antibody. WCL and Co-IP complexes were analyzed by western blot with antibodies against FLAG, ubiquitin. Parallel experiments using FLAG-RNF166-T157A as a control.
(TIF)

S8 Fig Effects of RNF166 and its mutant RNF166-T157A on the replication of rPDCoV M-K207R. **A,** Nucleotide sequencing of the recombinant virus rPDCoV M-K207R. **B-C,** The replication levels of rPDCoV M-K207R in wild-type (B) and RNF166 (KO) (C) IPI-2I cells expressing either RNF166 or RNF166-T157A, assessed by TCID_50_ assay. Data in panels B and C represent mean ± s.d. (n = 3). Statistical significance was determined by two-tailed Student’s t-test; *P < 0.05; **P < 0.01; ns, not significant.
(TIF)

S9 Fig HBP-driven UDP-GlcNAc production suppresses the replication of porcine coronaviruses. **A–C,** IPI-2I cells were infected with PEDV (A), TGEV (B), or SADS-CoV (C), respectively, and harvested at the indicated time points for RT-qPCR to measure the mRNA levels of GFAT and AGX. **D–F,** Viral replication of PEDV (D), TGEV (E), or SADS-CoV (F) in IPI-2I cells supplemented with or without UDP-GlcNAc, assessed by western blot. β-actin and GAPDH were used as internal controls for western blot and RT-qPCR respectively. Data in panels A–C represent mean ± s.d. (n = 3). Statistical significance was determined by two-tailed Student’s t-test; *P < 0.05; **P < 0.01; ns, not significant.
(TIF)

S10 Fig RNF166 fails to degrade the M proteins of human or murine coronaviruses. **A,** Conservation analysis of the M protein coding sequences among all known porcine coronaviruses, including PDCoV, PEDV, TGEV, PRCV, PHEV, and SADS-CoV. **B-D,** western blot analysis of M protein degradation in HEK293T cells co-expressing FLAG-RNF166 or FLAG-RNF166-T157A and HA-tagged M protein of SARS-CoV-2 (B), HCoV-229E (C), and MHV (D). **E,** Conservation analysis of the M protein coding sequences among PDCoV, HKU11, HCoV-229E, HCoV-NL63, HCoV-OC43, MHV, HCoV-HKU1, MERS-CoV, SARS-CoV, SARS-CoV-2, IBV, and TCoV.
(TIF)

S1 Table Primers used for plasmid construction. (XLSX)

S2 Table Primers used for siRNA interference. (XLSX)

S3 Table Primers used for Cas9-mediated sgRNA construction. (XLSX)

S4 Table Primers used for RT-qPCR. (XLSX)

S5 Table Subcellular localization of O-GlcNAcylation targets. (XLSX)

S1 Data Excel spreadsheet containing the underlying numerical data for Figs 1C-1I, 2A, 2B, 2G, 2F, 2H, 4A-4E, 6B, 6D, 6F, 6H, 7B-7F, S1A-1D, S1F, S1G, S1H, S2B, S2D, S2F, S4A-4E, S8B, S8C, S9A-C in separate sheets. (XLSX)
