# Supplementary figures and images for: O-GlcNAcylation licenses RNF166 to degrade the M protein of porcine coronaviruses
Source: PLoS Pathog. 2026 Jun 25;22(6):e1014301. doi: 10.1371/journal.ppat.1014301 (PMC13298740; doi:10.1371/journal.ppat.1014301)

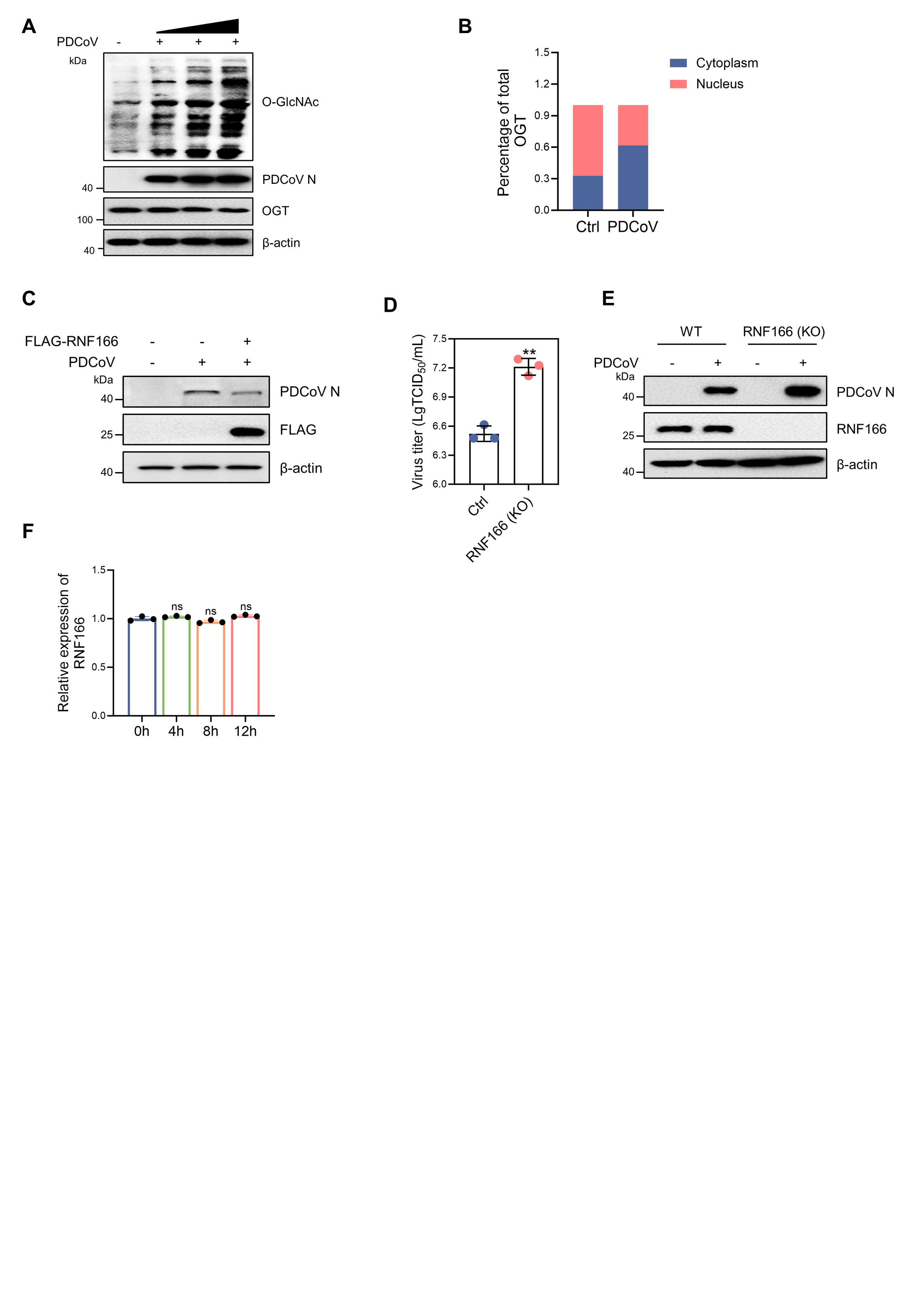

Supplement: S2 Fig — A, Cellular O-GlcNAcylation was assessed by western blot in IPI-2I cells at 12 hpi with various multiplicities of infection (MOIs). B, Grayscale intensity of OGT bands in the immunoblot shown in Fig 2D was quantified using ImageJ. C, Effect of RNF166 on PDCoV replication, as assessed in RNF166-overexpressing cells by western blot. D-E, Comparison of PDCoV replication between wild-type and RNF166 (KO) IPI-2I cells, assessed by TCID50 (D) assay and western blot (E). F, IPI-2I cells were infected with PDCoV (MOI = 2) and harvested at the indicated time points, followed by RT-qPCR analysis of RNF166 mRNA levels. Data in panels D and F represent mean ± s.d. (n = 3). Statistical significance was determined by two-tailed Student’s t-test; *P < 0.05; **P < 0.01; ns, not significant. (TIF) [file ppat.1014301.s002.tif]

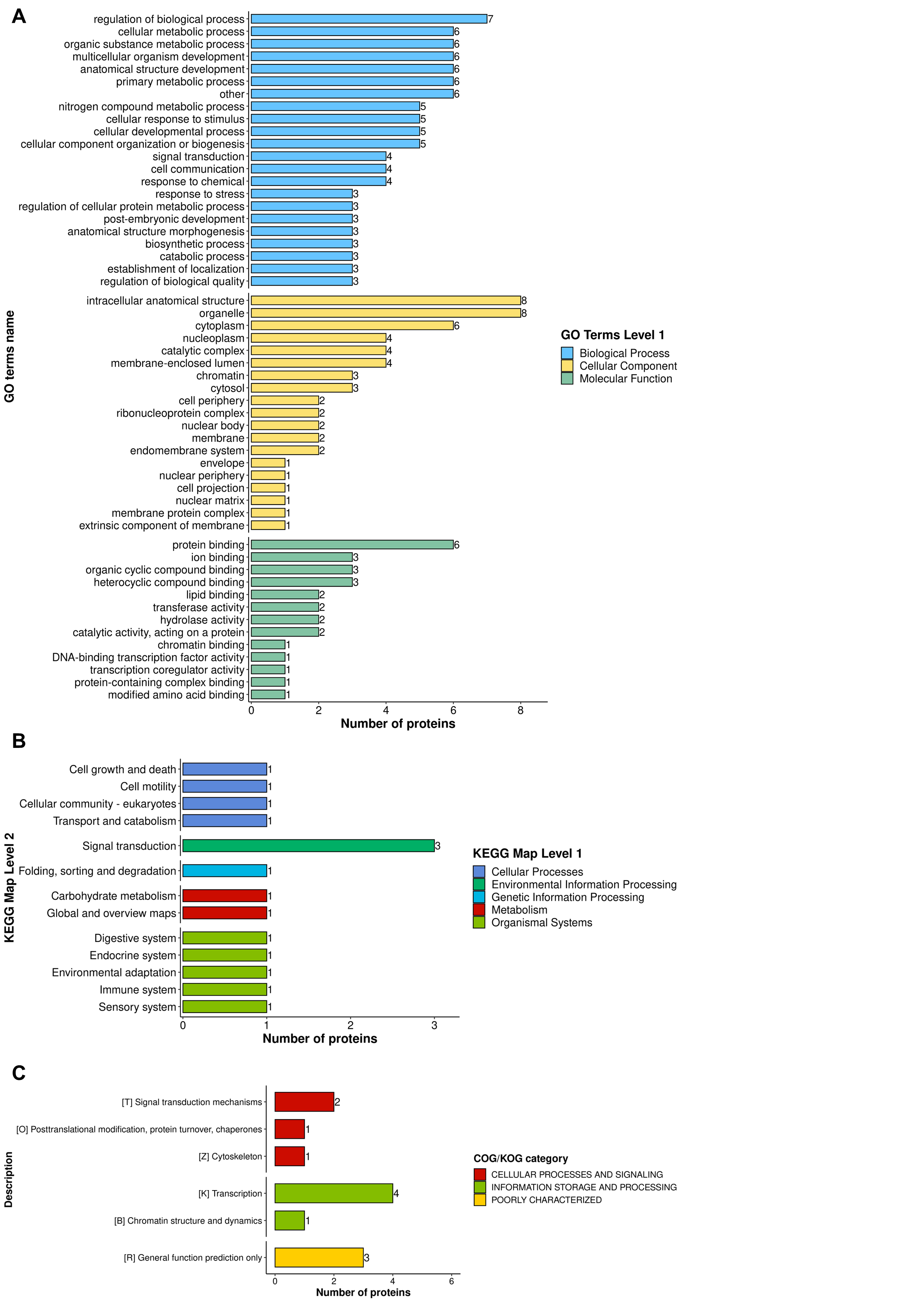

Supplement: S3 Fig — A–C, Identification of O-GlcNAcylated proteins by mass spectrometry from IPI-2I cells infected with PDCoV (MOI = 2) and harvested at 12 hpi, followed by enrichment analyses. (A) Gene Ontology (GO) enrichment. (B) Kyoto Encyclopedia of Genes and Genomes (KEGG) pathway. (C) Clusters of Orthologous Groups/Eukaryotic Orthologous Groups (COG/KOG). (TIF) [file ppat.1014301.s003.TIF]

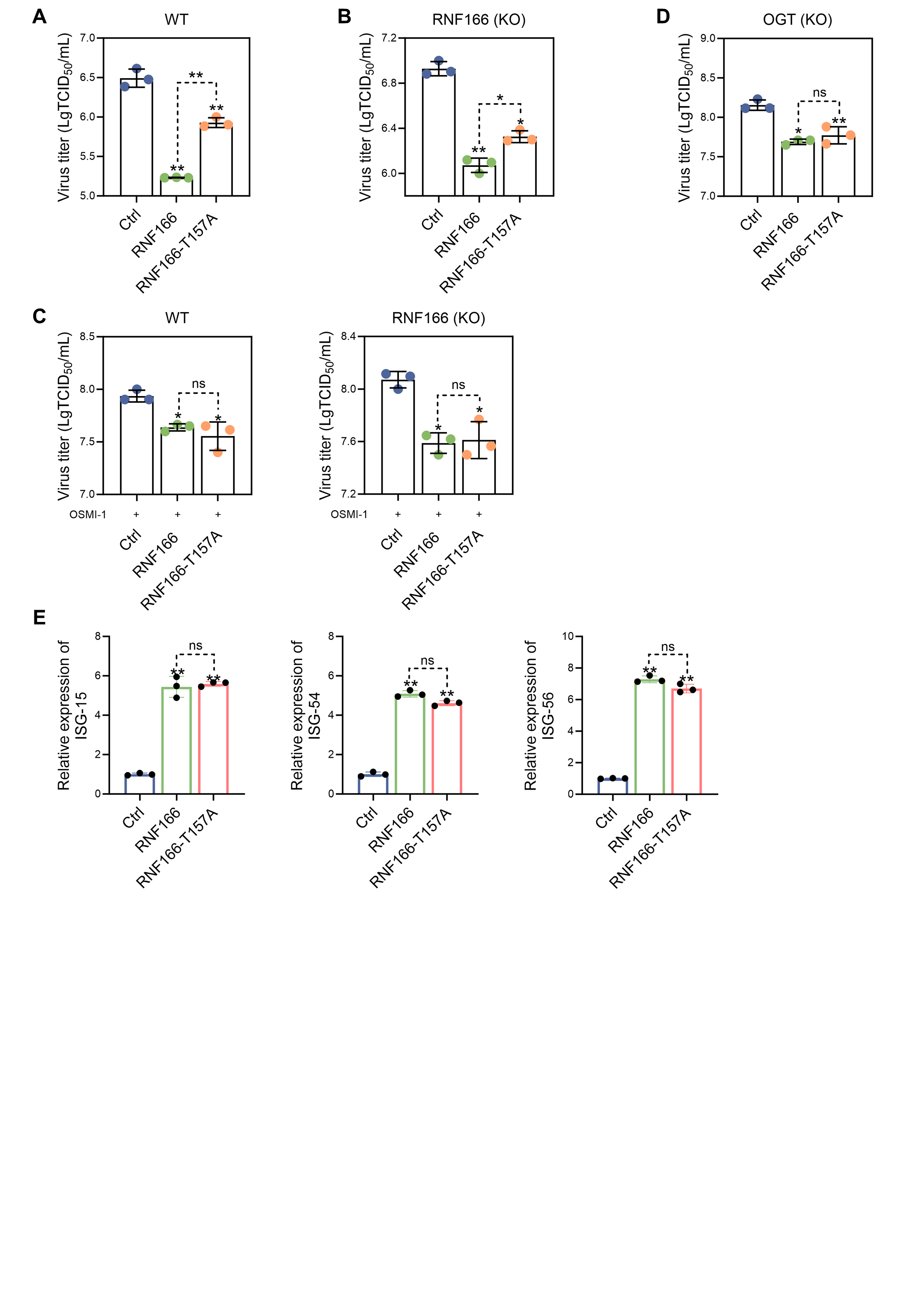

Supplement: S4 Fig — A-D, PDCoV replication was assessed by TCID50 assay in the following cells expressing either RNF166 or the mutant RNF166-T157A: (A) in wild-type IPI-2I cells. (B) in RNF166 (KO) IPI-2I cells. (C) in wild-type and RNF166 (KO) IPI-2I cells treated with OSMI-1 (50 μM). (D) in OGT (KO) IPI-2I cells. E, The mRNA levels of ISG15, ISG54 and ISG56 in HEK293T cells transfected with RNF166 or RNF166-T157A, assessed by RT-qPCR. Data in panels A–E represent mean ± s.d. (n = 3). Statistical significance was determined by two-tailed Student’s t-test; *P < 0.05; **P < 0.01; ns, not significant. (TIF) [file ppat.1014301.s004.TIF]

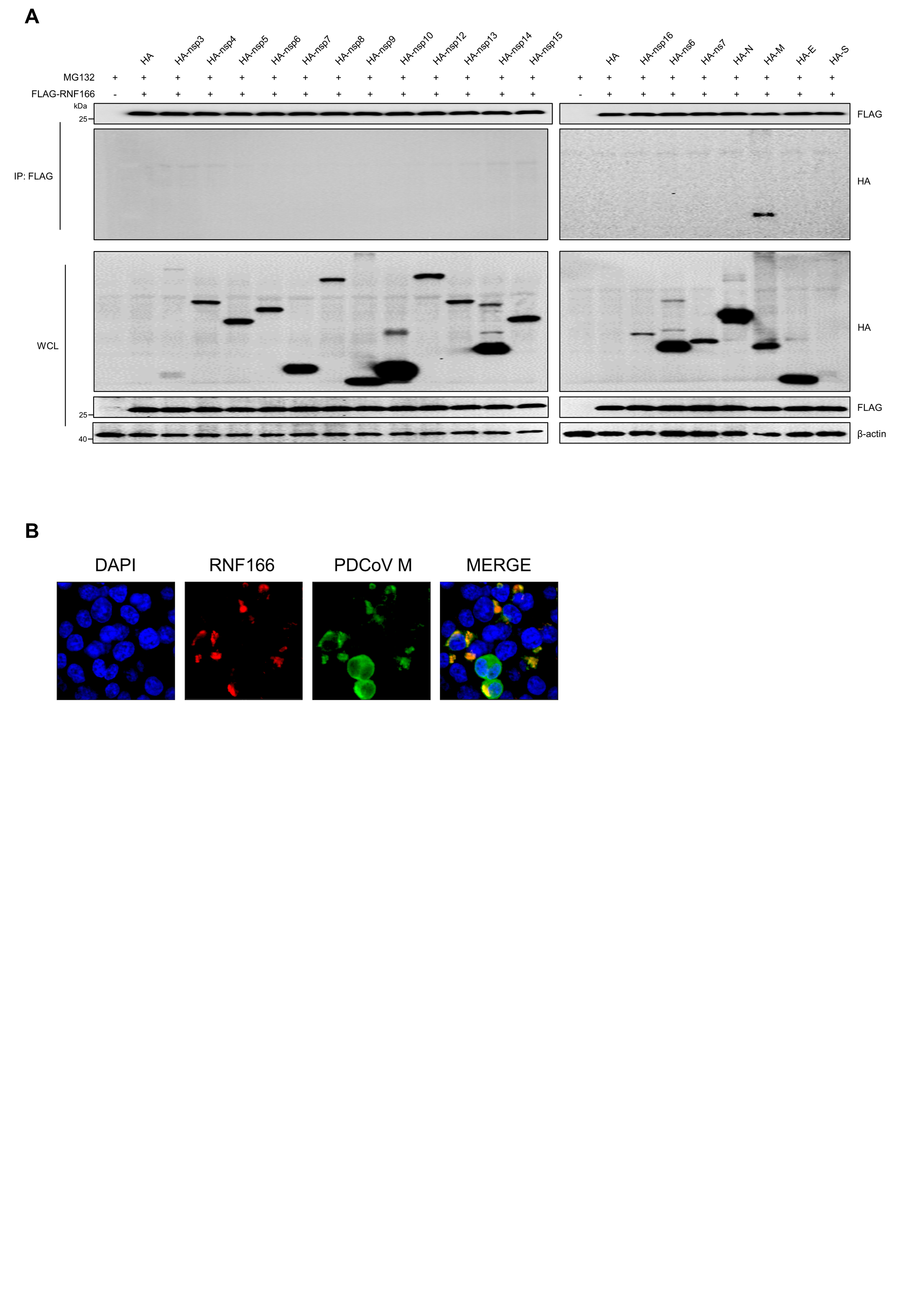

Supplement: S5 Fig — A, Screening for RNF166 partners that interact with PDCoV-encoded proteins in HEK293T cells, which were co-transfected with FLAG-RNF166 and HA-tagged PDCoV-encoded protein expression plasmids. Co-immunoprecipitation (Co-IP) was performed using an anti-FLAG antibody. Whole-cell lysates (WCL) and Co-IP complexes were immunoblotted with antibodies against HA and FLAG. B, IPI-2I cells were co-transfected with HA-tagged PDCoV M and FLAG-tagged RNF166, followed by indirect immunofluorescence analysis to assess their subcellular co-localization. PDCoV M is shown in green, RNF166 in red. Scale bar, 20 μm. (TIF) [file ppat.1014301.s005.TIF]

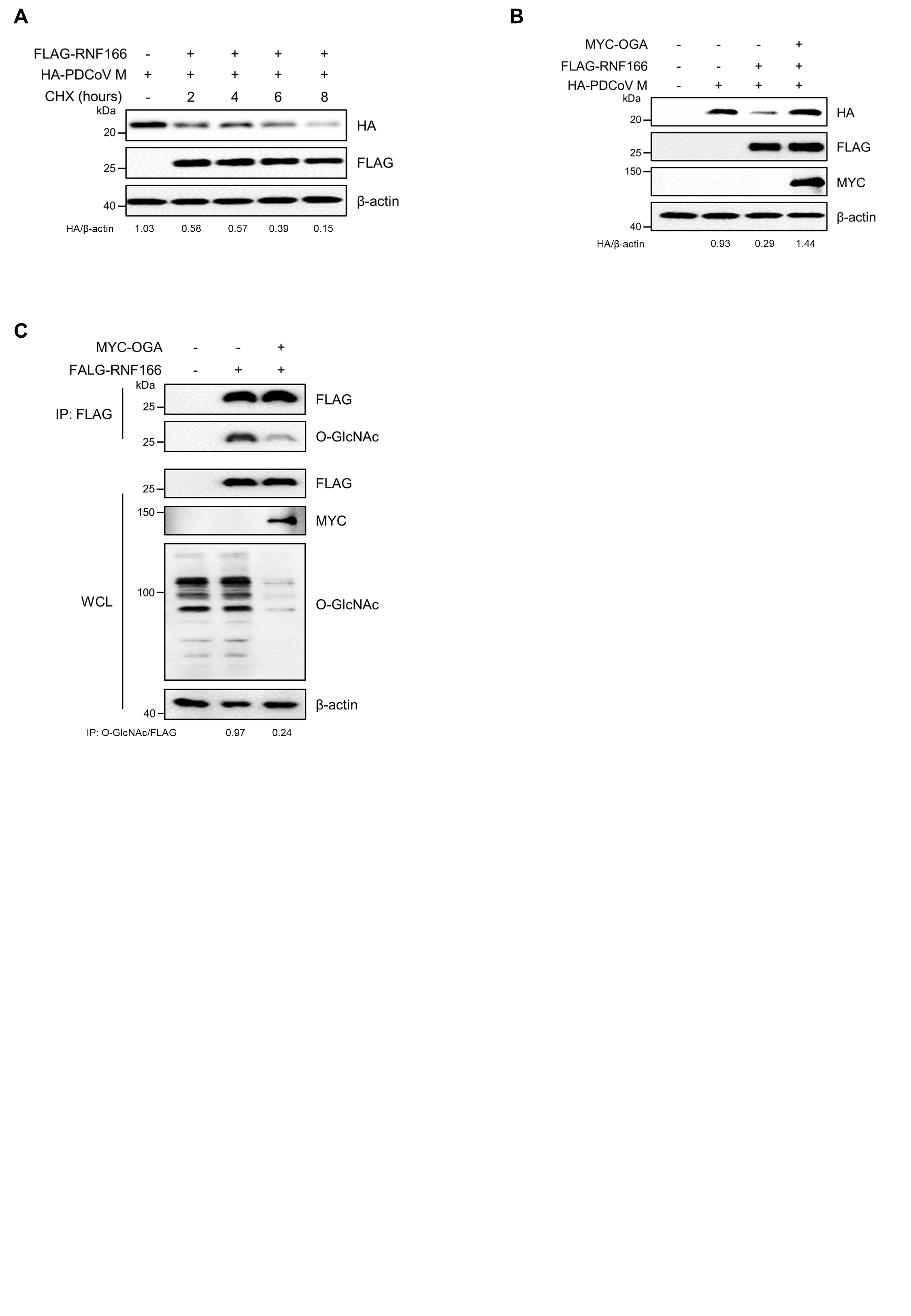

Supplement: S6 Fig — A, HEK293T cells were co-transfected with RNF166 and PDCoV M, followed by CHX treatment and PDCoV M protein levels were analyzed by western blot. B, HEK293T cells were co-transfected with MYC-OGA, FLAG-RNF166, and HA-PDCoV M, followed by western blot analysis of the effect of OGA on RNF166-mediated PDCoV M degradation. C, O-GlcNAcylation levels of RNF166 in HEK293T cells transfected with FLAG-RNF166 in the presence or absence of MYC-OGA, assessed by IP assay using an anti-FLAG antibody. WCL and FLAG immunoprecipitates were analyzed by western blot with antibodies against FLAG, MYC, O-GlcNAc, and β-actin. Western blot band intensities were quantified using ImageJ. (TIF) [file ppat.1014301.s006.tif]

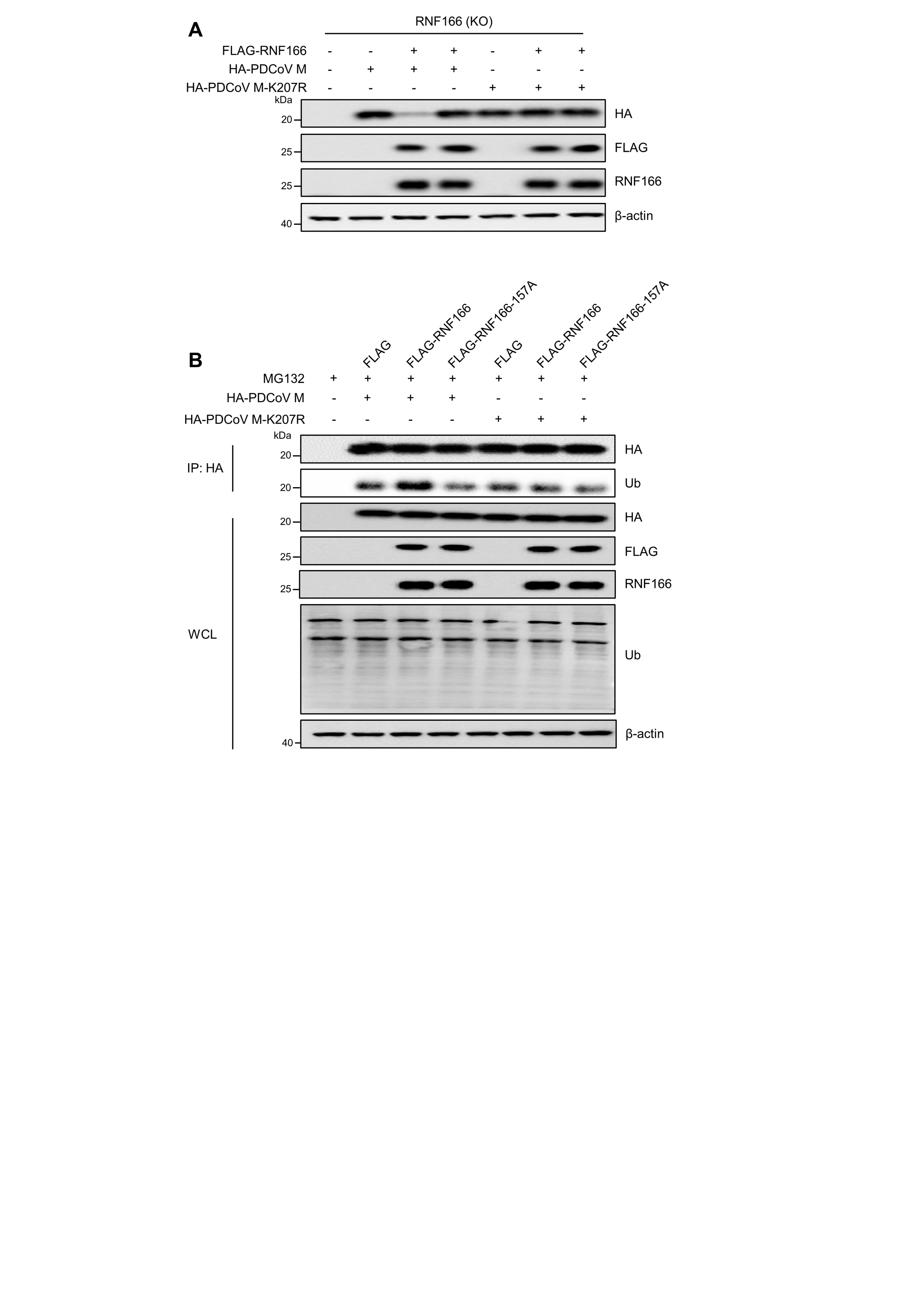

Supplement: S7 Fig — A, Effects of RNF166 on PDCoV M-K207R protein degradation in RNF166 (KO) IPI-2I cells co-transfected with FLAG-RNF166 and HA-PDCoV M-K207R. Parallel experiments using HA-PDCoV M as a control. B, Ubiquitination levels of wild-type HA-PDCoV M and its mutant HA-PDCoV M-K207R in HEK293T cells co-transfected with FLAG-RNF166, HA-PDCoV M or FLAG-RNF166, HA-PDCoV M-K207R, assessed by Co-IP assay using an anti-HA antibody. WCL and Co-IP complexes were analyzed by western blot with antibodies against FLAG, ubiquitin. Parallel experiments using FLAG-RNF166-T157A as a control. (TIF) [file ppat.1014301.s007.TIF]

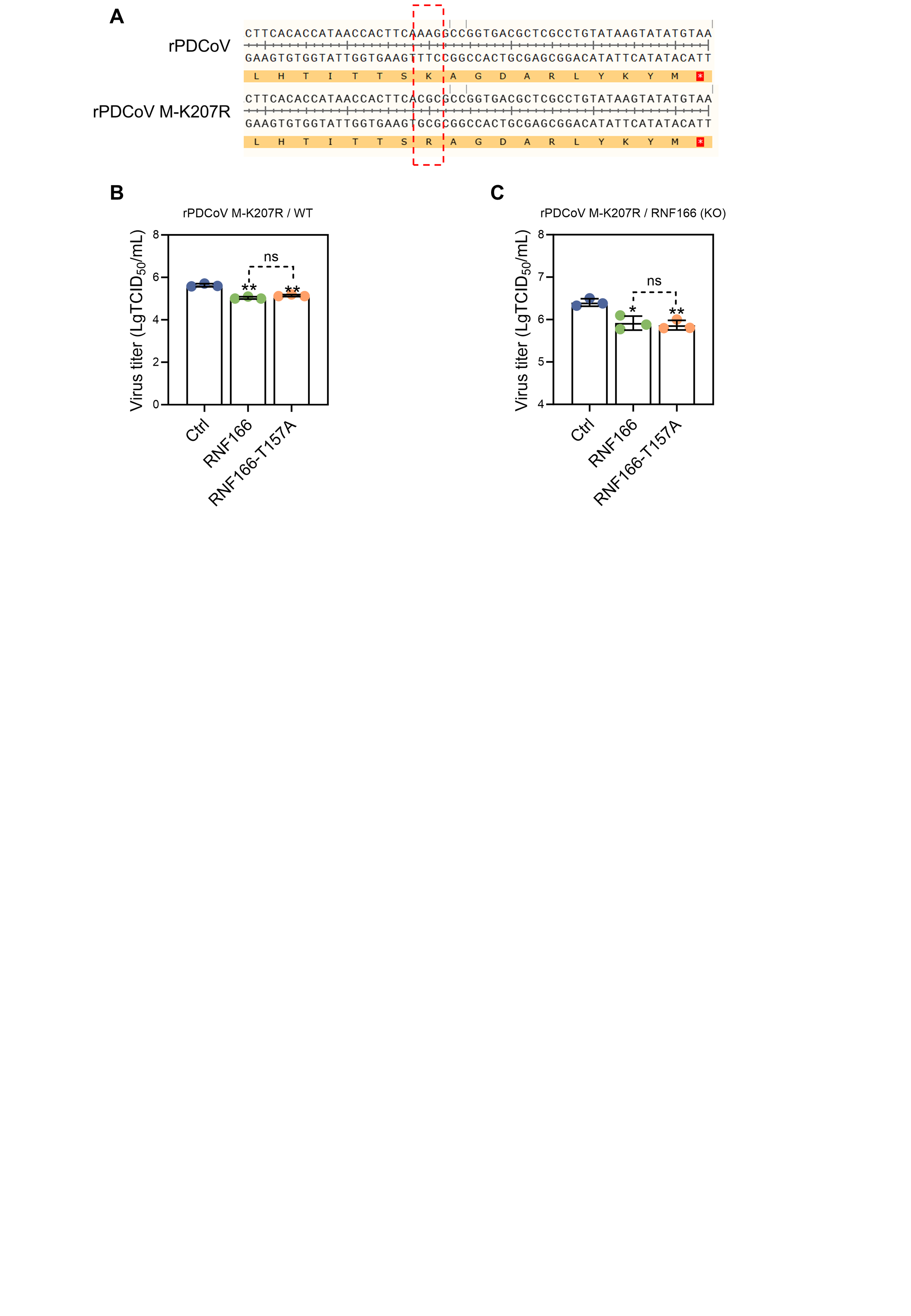

Supplement: S8 Fig — A, Nucleotide sequencing of the recombinant virus rPDCoV M-K207R. B-C, The replication levels of rPDCoV M-K207R in wild-type (B) and RNF166 (KO) (C) IPI-2I cells expressing either RNF166 or RNF166-T157A, assessed by TCID50 assay. Data in panels B and C represent mean ± s.d. (n = 3). Statistical significance was determined by two-tailed Student’s t-test; *P < 0.05; **P < 0.01; ns, not significant. (TIF) [file ppat.1014301.s008.TIF]

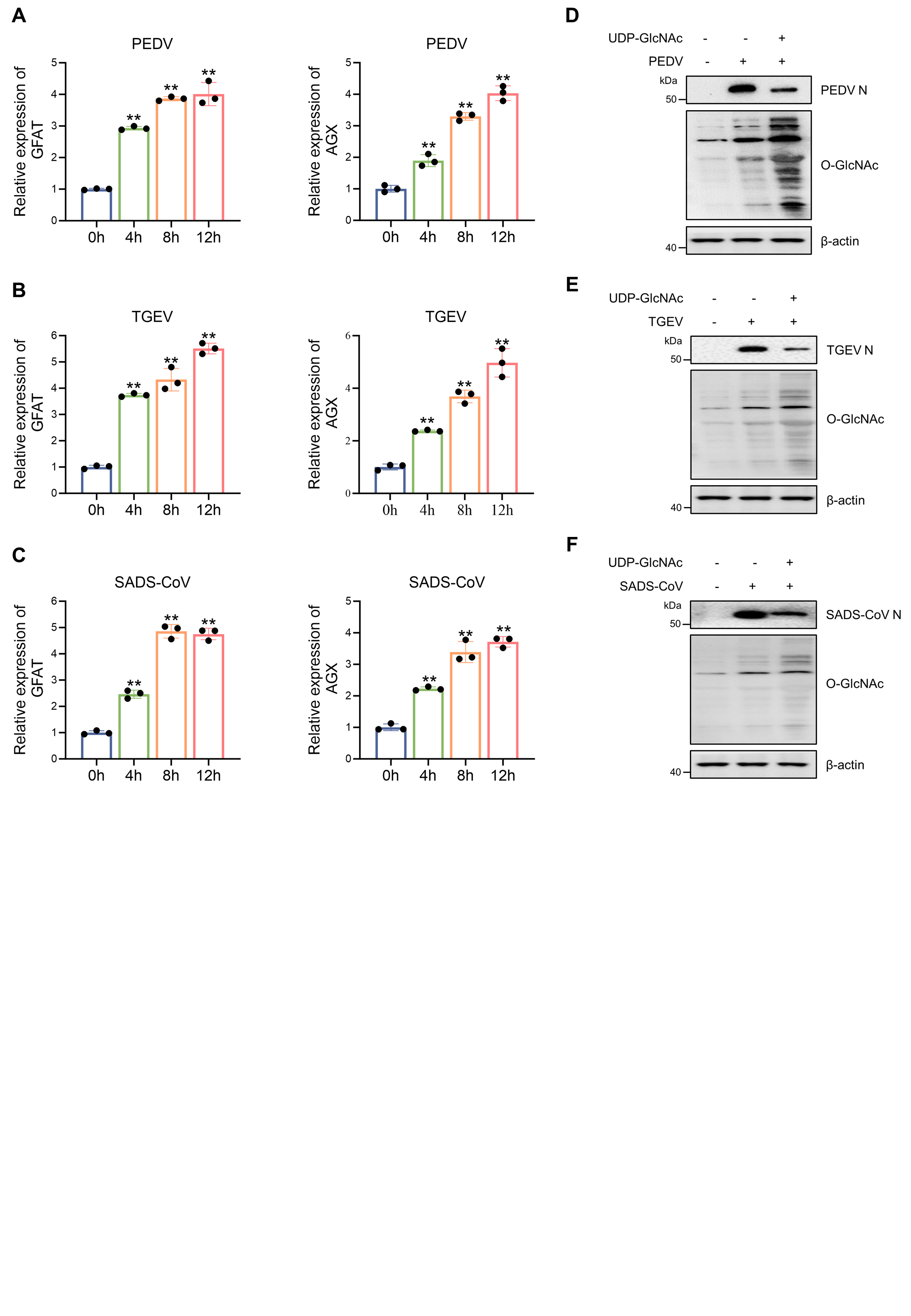

Supplement: S9 Fig — A–C, IPI-2I cells were infected with PEDV (A), TGEV (B), or SADS-CoV (C), respectively, and harvested at the indicated time points for RT-qPCR to measure the mRNA levels of GFAT and AGX. D–F, Viral replication of PEDV (D), TGEV (E), or SADS-CoV (F) in IPI-2I cells supplemented with or without UDP-GlcNAc, assessed by western blot. β-actin and GAPDH were used as internal controls for western blot and RT-qPCR respectively. Data in panels A–C represent mean ± s.d. (n = 3). Statistical significance was determined by two-tailed Student’s t-test; *P < 0.05; **P < 0.01; ns, not significant. (TIF) [file ppat.1014301.s009.TIF]

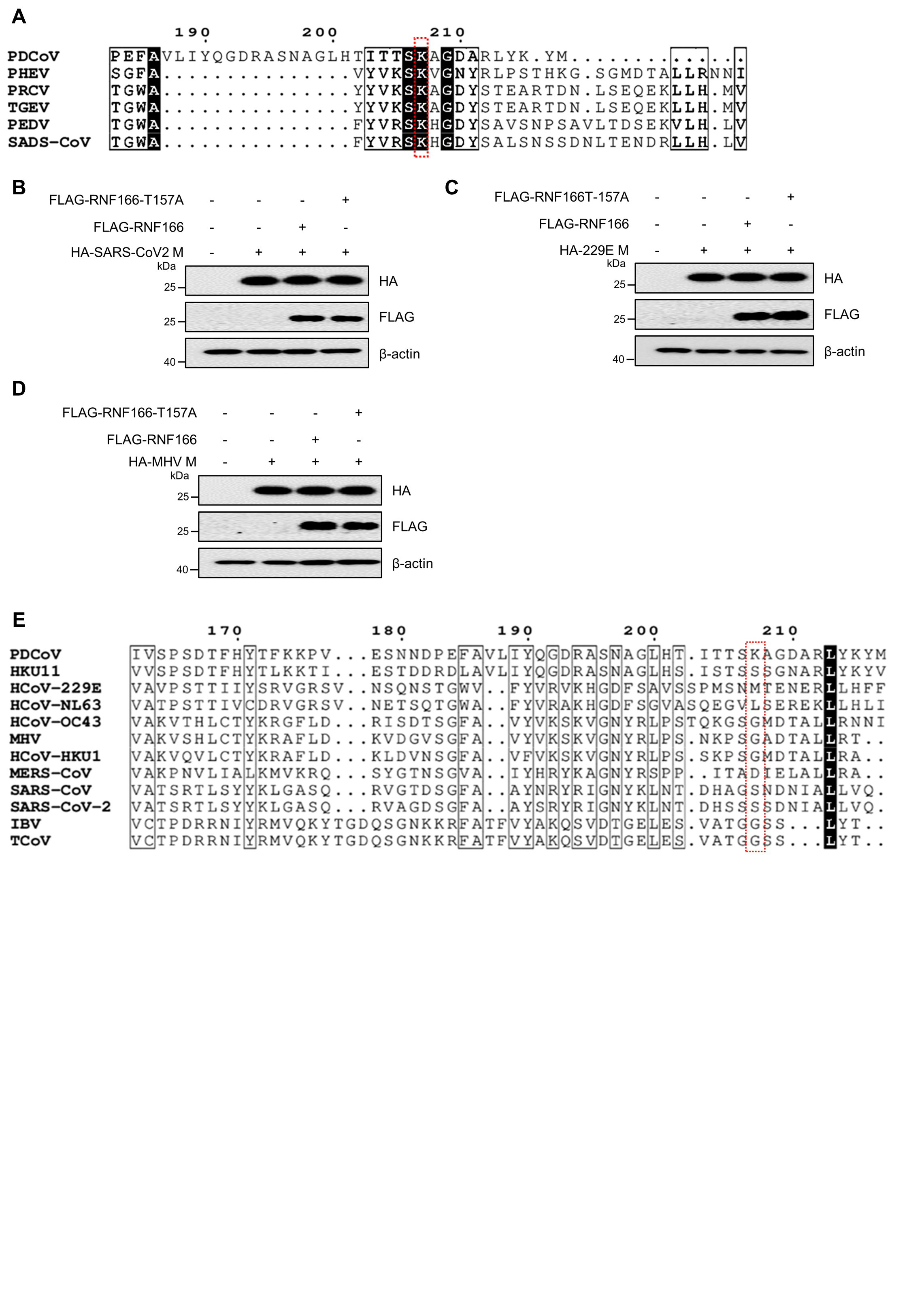

Supplement: S10 Fig — A, Conservation analysis of the M protein coding sequences among all known porcine coronaviruses, including PDCoV, PEDV, TGEV, PRCV, PHEV, and SADS-CoV. B-D, western blot analysis of M protein degradation in HEK293T cells co-expressing FLAG-RNF166 or FLAG-RNF166-T157A and HA-tagged M protein of SARS-CoV-2 (B), HCoV-229E (C), and MHV (D). E, Conservation analysis of the M protein coding sequences among PDCoV, HKU11, HCoV-229E, HCoV-NL63, HCoV-OC43, MHV, HCoV-HKU1, MERS-CoV, SARS-CoV, SARS-CoV-2, IBV, and TCoV. (TIF) [file ppat.1014301.s010.tif]
